# Ca_4_Sb_2_O and Ca_4_Bi_2_O: two promising mixed-anion thermoelectrics†

**DOI:** 10.1039/d1ta03649a

**Published:** 2021-08-02

**Authors:** Warda Rahim, Jonathan M. Skelton, David O. Scanlon

**Affiliations:** Department of Chemistry, University College London 20 Gordon Street London WC1H 0AJ UK d.scanlon@ucl.ac.uk; Thomas Young Centre, University College London Gower Street London WC1E 6BT UK; Department of Chemistry, University of Manchester Oxford Road Manchester M13 9PL UK; Diamond Light Source Ltd., Diamond House, Harwell Science and Innovation Campus Didcot Oxfordshire OX11 0DE UK

## Abstract

The environmental burden of fossil fuels and the rising impact of global warming have created an urgent need for sustainable clean energy sources. This has led to widespread interest in thermoelectric (TE) materials to recover part of the ∼60% of global energy currently wasted as heat as usable electricity. Oxides are particularly attractive as they are thermally stable, chemically inert, and formed of earth-abundant elements, but despite intensive efforts there have been no reports of oxide TEs matching the performance of flagship chalcogenide materials such as PbTe, Bi_2_Te_3_ and SnSe. A number of ternary X_4_Y_2_Z mixed-anion systems, including oxides, have predicted band gaps in the useful range for several renewable-energy applications, including as TEs, and some also show the complex crystal structures indicative of low lattice thermal conductivity. In this study, we use *ab initio* calculations to investigate the TE performance of two structurally-similar mixed-anion oxypnictides, Ca_4_Sb_2_O and Ca_4_Bi_2_O. Electronic-structure and band-alignment calculations using hybrid density-functional theory (DFT), including spin–orbit coupling, suggest that both materials are likely to be p-type dopable with large charge-carrier mobilities. Lattice-dynamics calculations using third-order perturbation theory predict ultra-low lattice thermal conductivities of ∼0.8 and ∼0.5 W m^−1^ K^−1^ above 750 K. Nanostructuring to a crystal grain size of 20 nm is predicted to further reduce the room temperature thermal conductivity by around 40%. Finally, we use the electronic- and thermal-transport calculations to estimate the thermoelectric figure of merit *ZT*, and show that with p-type doping both oxides could potentially serve as promising earth-abundant oxide TEs for high-temperature applications.

## Abstract 2

Using first-principles modelling we predict Ca_4_Sb_2_O and Ca_4_Bi_2_O to have ultra-low lattice thermal conductivity, making them promising earth-abundant oxide thermoelectrics.

## Graphical abstract

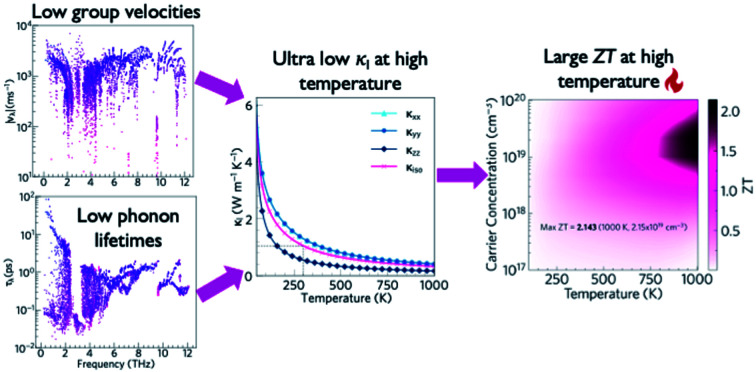

## 1 Introduction

Recovering the ∼60% of global energy currently wasted as heat is critical to tackling the environmental and economic issues emerging from climate change. Despite years of research in the field, current state-of-the-art thermoelectrics (TEs) are not suitable for widespread commercial use due to either a low efficiency or compositions based on rare or toxic elements such as lead and tellurium.^1,2^ Their use has therefore been largely confined to niche applications, *e.g.* in batteries to power space equipment or in electronic cooling.^3,4^ The thermoelectric efficiency of a material is commonly defined using the dimensionless figure of merit *ZT* = *S*^2^*σT*/*κ* where *S* is the Seebeck coefficient, *σ* is the electrical conductivity, *T* is the absolute temperature, and *κ* is the sum of the electronic and lattice thermal conductivity *κ*_e_ + *κ*_l_. One of the biggest challenges in the field is thus the discovery of new materials with the required balance of physical properties to obtain a high *ZT*.^5^ The *ZT* can be enhanced either by optimising the electronic transport properties or by minimizing the lattice thermal conductivity. Doping and band-engineering techniques, either to tune the carrier effective masses or to increase the valley degeneracy, are often employed to optimise the electrical properties.^6–10^ The interdependency of *S*, *σ* and *κ*_e_ however makes this quite a complex task. The dominant thermal transport mechanism in semiconductors is through the phonons (*i.e. κ*_l_), and high-performance TEs therefore tend to be materials with low intrinsic lattice thermal conductivity. To reduce *κ*_l_ further, nanostructuring, for example by grain-size engineering, can be employed to scatter phonons with medium and long mean free paths to increase the overall thermal resistance.^1,11–13^ However, this usually fails to scatter the phonons with short and long mean free paths, which has led to the concept of multi-scale “hierarchical” materials-engineering strategies to scatter phonons on different length scales.^14^

A viable mass-market thermoelectric needs to be cost-effective, stable under the temperature gradients in operating devices, should maintain a high average *ZT* over a wide operating temperature range, and should be chemically inert to avoid the risk of device deterioration and to avoid problems with end-of-life disposal. The current industry-standard thermoelectric materials, which include PbTe, PbSe and Bi_2_Te_3_, contain rare or toxic elements.^15–17^ However, many promising alternatives have failed to succeed them due to poor thermal stability.^18,19^ Given these requirements, there has been much effort focused toward oxides, as these often show the required thermal and chemical stability, are easy to synthesise, and are formed of earth-abundant and non-toxic elements.^20,21^ Though there has been considerable progress in this area, the maximum *ZT* of even the best materials remain quite low compared to other thermoelectrics, typically due to a moderate electrical conductivity and/or a high thermal conductivity.^22,23^ Recently, we reported the promising thermoelectric properties of the room temperature polymorph of Bi_2_Sn_2_O_7_, which we found to have an ultralow intrinsic lattice thermal conductivity of ∼0.4 W m^−1^ K^−1^ and a *ZT* of 0.18 with n-type doping.^24^ Although this *ZT* is the highest ever obtained for an oxide material at room temperature, Bi_2_Sn_2_O_7_ may not be suitable for high-temperature applications as it undergoes a phase transition above 390 K.

It is also noteworthy that both n- and p-type materials with high thermoelectric figures of merit are needed to form a TE couple, and oxides are in general usually not p-type dopable.^25^ However, systems which contain O^2−^ and another anion, for example BiCuOSe, show good p-type dopability and have emerged as promising p-type thermoelectrics.^26–28^ In a recent computational study,^29^ X_4_Y_2_Z mixed-anion compounds (X = Mg, Ca, Sr, Ba; Y = P, As, Sb, Bi; and Z = S, Se, Te) have been predicted to possess band gaps in the range suitable for many renewable-energy applications including as thermoelectrics. The study also revealed high band degeneracies, indicative of high power factors, and the complex crystal structures were found to yield low lattice thermal conductivities, with the room temperature *κ*_l_ of Ba_4_Sb_2_Se being comparable to the flagship thermoelectric SnSe.^30,31^ Moreover, quite recently ferroelectric and antiferroelectric behaviours and applications to ferroelectric photovoltaics have been reported for this family of anti-Ruddlesden–Popper phases.^32^ Inspired by this, we have investigated the p-type thermoelectric properties of two experimentally-reported mixed-anion oxypnictides with similar composition, *viz.* Ca_4_Sb_2_O and Ca_4_Bi_2_O.

**Fig. 1:**
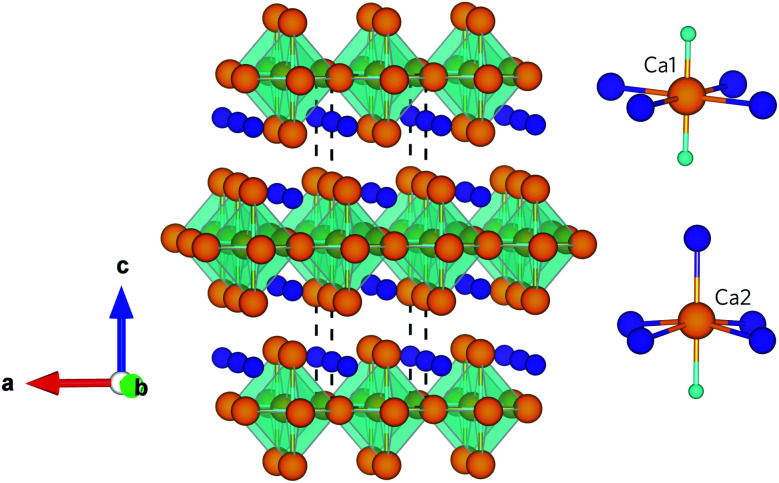
Crystal structure of Ca_4_Sb_2_O and Ca_4_Bi_2_O showing the O-centred Ca octahedra stacked along the crystallographic *c* axis. The insets show the coordination polyhedra around the two distinct Ca atoms. The atoms are coloured as follows: Ca – orange, O – cyan, Sb/Bi – purple. The images were generated using the VESTA software.^33^

Ca_4_Sb_2_O and Ca_4_Bi_2_O are isotypes of the anti-K_2_NiF_4_ structure type, belonging to the series of compounds A_4_M_2_O (A = Ca, Sr, Ba; M = P, As, Sb, Bi).^34–36^ Both compounds crystallise into a body-centred tetragonal structure with the *I*4/*mmm* space group (139), which is illustrated in Fig. 1. The crystal structure can be considered as an infinite network of layers comprising oxygen-centred Ca octahedra stacked along the *c*-axis in an ABAB fashion. The structure consists of two crystallographically unique Ca sites with different coordination environments: the Ca1 site is surrounded by four coplanar M ions (M = Sb/Bi) and two O ions, and the Ca2 site is surrounded by four equidistant M ions, one apical M ion and one O ion. Ca2 forms a stronger (shorter) bond to the apical M anion compared to those in the equatorial plane, which results in the Ca2–O bond being longer than the Ca1–O bond. This complex crystal structure and inhomogenous bonding could lead to large phonon anharmonicity and thus low lattice thermal conductivity. Moreover, the presence of the heavy elements Sb and Bi will result in low group velocities and weak chemical bonding within the structures, features which both often predicate low *κ*_l_. In addition, the low-frequency vibrations of these weakly-bonded heavy atoms are likely to enable a high density of scattering channels for the acoustic phonon modes which, provided these modes make the largest contribution to the *κ*_l_, should result in enhanced thermal resistance.

In this work, we investigate the electronic structure and transport properties using hybrid density-functional theory (DFT) with spin–orbit coupling and we study the lattice thermal transport using lattice-dynamics calculations. We find good agreement between our electronic density of states (DoS) calculations and previous work by Xia *et al.*^36^ with the valence band maxima and conduction band minima consisting predominantly of Sb/Bi p-states and Ca 3d states respectively. Both materials are found to have favourable electrical properties for thermoelectric applications, with low carrier effective masses and suitable band gaps, and an assessment of the valence band energies indicate native p-type semiconducting behaviour. Thermal-conductivity calculations show that the acoustic modes and low-frequency optic modes primarily responsible for the bulk of thermal transport in both structures are short-lived, leading to ultra-low *κ*_l_. These short lifetimes are found to arise from both a high density of low-frequency optic phonon modes, providing a large number of energy-conserving scattering channels, together with strong phonon–phonon interactions. To the best of our knowledge this represents the first comprehensive investigation of the electronic and thermal transport properties of Ca_4_Sb_2_O and Ca_4_Bi_2_O, and the fundamental understanding from this modelling study will help to provide guiding principles to identify and improve oxide-based TEs suitable for widespread use.

## 2 Computational methodology

Our calculations were performed within the framework of pseudopotential plane-wave DFT as implemented in the Vienna Ab initio Simulation Package (VASP) code.^37–40^ Projector augmented wave (PAW) pseudopotentials^41,42^ were used to describe the core electrons, with Ca 3s^2^3p^6^4s^2^, Sb 5s^2^5p^3^, Bi 5d^10^6s^2^6p^3^ and O 2s^2^2p^4^ electrons treated as valence. Explicit convergence testing with criteria of 5 meV per atom for the plane-wave kinetic-energy cutoff and 1 meV per atom for the *k*-point sampling mesh suggested a 400 eV cutoff for both structures and *Γ*-centred *k*-point meshes with 5 × 5 × 7 and 5 × 5 × 6 subdivisions for Ca_4_Sb_2_O and Ca_4_Bi_2_O respectively (Fig. S1†). During the geometry optimisations the cutoff was increased to 520 eV to avoid Pulay stress.^43^ The cell parameters and atomic positions were fully relaxed using the variant of the Perdew–Burke–Ernzerhof (PBE)^44^ generalised-gradient approximation functional for solids (PBEsol).^45^ The optimisations were performed to a force tolerance < 1 × 10^−4^ eV Å^−1^.

Electronic-structure calculations were performed using the screened hybrid HSE06 functional^46,47^ including spin–orbit coupling (SOC) effects. The effective masses at the band edges were calculated according to:1
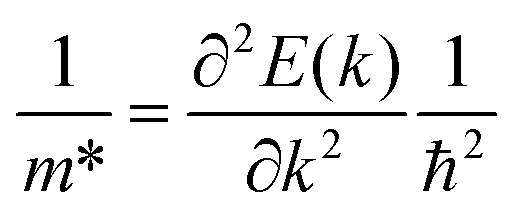
where *E*(*k*) is the band energy as a function of the electron wavevector *k* and *ℏ* is the reduced Planck's constant.

The electronic transport properties were calculated using the AMSET software package.^48^ The code uses the momentum relaxation time approximation (MRTA)^49^ to calculate the scattering rates and carrier mobilities, and has demonstrated excellent agreement both to experimental measurements of the electron mobilities and Seebeck coefficients of several semiconductors and to highly-accurate theoretical calculations using the electron-phonon Wannier approach.^48,50–52^ Unlike the widely-used BoltzTraP approach,^53^ this method does not assume a constant relaxation time and instead estimates one by considering a range of scattering processes. We considered three scattering processes in this work, *viz.* the acoustic deformation potential (ADP), ionized impurity (IMP), and polar-optical phonon (POP) scattering. The characteristic scattering *τ*_e_ is then calculated following Matthiessen's rule:2
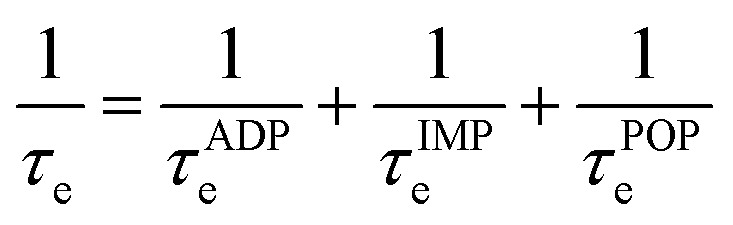


The mode dependent scattering rates for each process are obtained within the Born approximation using common materials parameters, with differential scattering rates from state |*n***k**〉 to state |*m***k** + **q**〉 calculated using Fermi's golden rule:^48^3
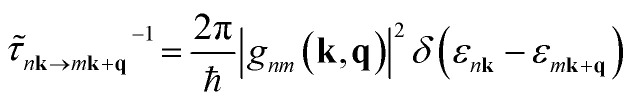
where *ε*_*n***k**_ represents the energy of the state |*n***k**〉, and *g*_*nm*_(**k**,**q**) is the matrix element for scattering from state |*n***k**〉 into state |*m***k** + **q**〉.

The material parameters required as input to the method, including high-frequency and static dielectric constants, elastic constants, phonon frequencies and deformation potentials, were all obtained using first-principles calculations. To obtain the high-frequency dielectric constants and deformation potentials, calculations were performed using HSE06 with SOC. The ionic dielectric constants, elastic constants and the “effective polar phonon frequency” were obtained using density-functional perturbation theory (DFPT)^54^ with the PBEsol functional. The transport properties need to be converged with respect to an interpolation factor which controls the number of *k*-points in the interpolated band structures. Convergence results are provided in Fig. S2 and S3,† based on which a dense Fourier interpolated mesh of 69 × 69 × 93 was chosen (the original input *k*-point mesh used for the electronic band structures computed using DFT was 14 × 14 × 18).

Harmonic lattice dynamics calculations were performed using the finite-displacement method^55,56^ implemented in the Phonopy package.^57,58^ The accuracy of phonon frequencies is dependent on the range of the real-space interatomic force constants (IFCs) evaluated with the chosen supercell expansion. We, therefore checked the convergence of the harmonic phonon dispersions with respect to the supercell size. Although the dispersions were found to be reasonably converged with a small 84-atom cubic supercell created from the primitive cell (Fig. S4†), for greater accuracy we opted to use a 4 × 4 × 4 expansion of the primitive with 448 atoms. The dispersion curves of the two structures were evaluated along a path passing through the high-symmetry wavevectors in the *I*4/*mmm* Brillouin zones. To obtain atom-projected phonon density of states (pDoS) curves, Fourier interpolation was used to obtain frequencies on a uniform *Γ*-centred *q*-point mesh with 48 × 48 × 48 subdivisions.

The lattice thermal conductivity *κ*_l_ was computed within the single-mode relaxation time approximation (SM-RTA) as a sum of contributions from individual phonon modes *λ* according to:4
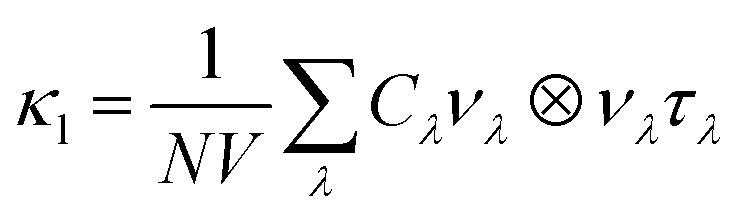
where *C*_*λ*_ are the modal heat capacities, *ν*_*λ*_ are the group velocities, *τ*_*λ*_ are the lifetimes, *V* is the volume of the unit cell, and *N* is the number of wavevectors used for the *BZ* integration (equivalent to the number of unit cells in the crystal).

The *C*_*λ*_ and *ν*_*λ*_ are obtained within the harmonic approximation. The *τ*_*λ*_ are obtained as the inverse of the phonon linewidths *Γ*_*λ*_, with *τ*_*λ*_ = 1/(2*Γ*_*λ*_). The *Γ*_*λ*_ are calculated using the imaginary part of the phonon self-energy, which can be computed perturbatively to third order by considering energy- and momentum-conserving three-phonon scattering processes.^59^ This requires the third-order force constants, which we computed using the finite-displacement approach implemented in the Phono3py code.^59^ This perturbative treatment of the linewidths/lifetimes has been used to successfully model the *κ*_l_ of many materials.^60–63^

Due to the larger number of pairwise interactions that must be considered for the third-order IFCs, the calculation of the phonon linewidths is 1–2 orders of magnitude more expensive than obtaining the second order IFCs. Moreover, it has been shown that the real-space range of the third-order IFCs is generally smaller than the second-order IFCs.^64^ The third-order IFCs were therefore calculated using 84-atom cubic supercell expansions of the primitive cells using the transformation matrix shown in eqn (5).5
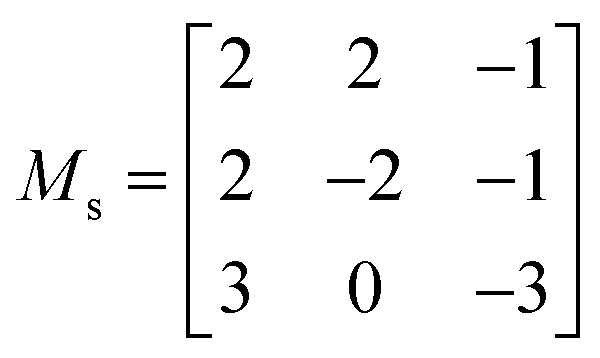


The PBEsol exchange–correlation functional was used for the force calculations, as it has shown to give a good description of lattice dynamics.^65–69^ The cutoff of 520 eV used for structural relaxation was also used for the force calculations to ensure accurate forces. SOC effects were not taken into account as they often have a negligible impact on phonon frequencies but greatly increase the computational cost. The *k*-point meshes used for the primitive cells were scaled appropriately for the supercell calculations. The *κ*_l_ of both structures were computed using *Γ*-centred *q*-point meshes with 15 × 15 × 15 subdivisions (Fig. S5†), using the linear tetrahedron method for the BZ integration.^70–72^

## 3 Results and discussion

### 3.1 Equilibrium geometry and electronic structure

A comparison of the calculated and experimental lattice parameters for the two systems is given in Table 1. The lattice parameters are systematically underestimated with PBEsol, consistent with previous trends,^73,74^ but are within 1.5% of the experimental structures, indicating that the PBEsol functional provides a good description of the equilibrium geometries.^67,68,74–77^

**Table 1:** Calculated lattice parameters of Ca_4_Sb_2_O and Ca_4_Bi_2_O. The % differences to the experimental structures^34,36^ are given in parentheses

Compound	*a* (Å)	*c* (Å)
Ca_4_Sb_2_O	4.648	16.141
	(−0.62%)	(−1.23%)
Ca_4_Bi_2_O	4.697	16.369
	(−0.46%)	(−0.82%)

Selected interatomic distances in Ca_4_Sb_2_O and Ca_4_Bi_2_O are given in Table 2. The bond lengths are relatively longer in Ca_4_Bi_2_O compared to Ca_4_Sb_2_O due to the larger ionic radius of Bi.

**Table 2:** Selected interatomic distances (Å) in Ca_4_Sb_2_O and Ca_4_Bi_2_O. The % differences to the experimental structures^34,36^ are given in parentheses

Bonds	Ca_4_Sb_2_O	Ca_4_Bi_2_O
Ca1–O1	2.324 (−0.6%)	2.349 (−0.5%)
Ca2–O1	2.661 (−2.4%)	2.664 (−2.2%)
Ca1–Sb1/Bi1	3.236 (−0.6%)	3.292 (−0.3%)
Ca2–Sb1/Bi1	3.312 (−0.8%)	3.341 (−0.7%)
Ca2–Sb1/Bi1	3.158 (−0.7%)	3.214 (−0.2%)
Ca1–Ca1	3.287 (−0.6%)	3.322 (−0.4%)
Ca1–Ca2	3.533 (−1.6%)	3.552 (−1.4%)

**Fig. 2:**
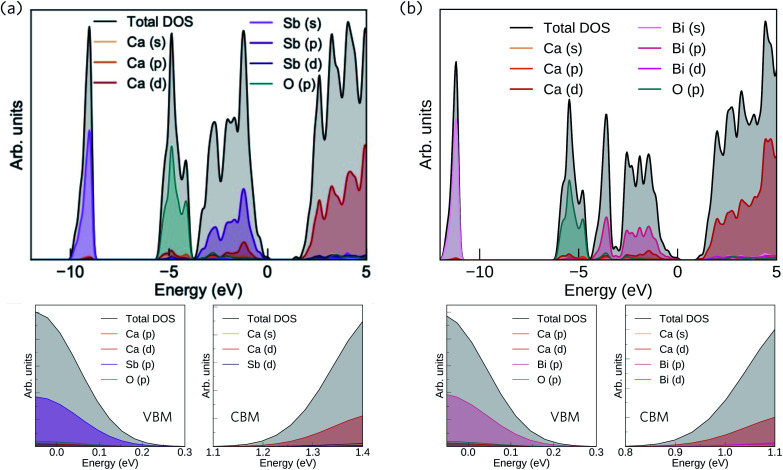
Calculated total and partial electronic density of states (DoS) curves of Ca_4_Sb_2_O (a) and Ca_4_Bi_2_O (b), both obtained at the HSE06 + SOC level of theory. The top of the valence band (VB) is set to 0 eV. The subplots below each DoS show expanded views of the valence band maxima (VBM) and conduction band minima (CBM). The plots were generated using the Sumo code, an open source command-line plotting tool for first-principles calculations.^78^

The calculated total and partial electronic density of states (DoS) of Ca_4_Sb_2_O and Ca_4_Bi_2_O are shown in Fig. 2 together with expanded views of the valence band maxima (VBM) and conduction band minima (CBM).

From 0 to −4 eV the valence band (VB) of Ca_4_Sb_2_O is mainly dominated by Sb 5p states, with some mixing with Ca 3d and 4p, and O 2p states. From −4 to −6 eV, the VB consists predominantly of O 2p states with small contributions from Ca 4s, 4p and 3d, and Sb 5p states. Towards the valence band maximum (VBM) at 0 eV the contribution from Sb 5p states increases and they undergo hybridisation with O 2p and Ca 4p and 3d states. The conduction band minimum (CBM) consists primarily of Ca d states, with a small amount of mixing with Ca s and Sb d states. Away from the CBM, the conduction band again consists mainly of Ca d states, with small contributions from Sb s, p and d, and O p states.

For Ca_4_Bi_2_O, the top of the VB from 0 to −2 eV is dominated by Bi 6p states with some contribution from Ca 3d states. The region of the VB from −2 and −4 eV is again mainly dominated by Bi 6p states, but this time mixed with Ca 4p and 3d, and O 2p states. (Around −3 eV the DoS predominantly consists of Bi 6p states only.) From around −4.5 eV to −6 eV, a large density of O 2p states is observed with a small of mixing with Ca 4s, 4p and 3d, and Bi 6p states. The VBM consists of Bi 6p states showing minimal hybridisation with O 2p and Ca 4p and 3d states. The CBM is again mainly dominated by Ca d states and shows a small amount of mixing with Bi p and d, and Ca s states. Again similarly to Ca_4_Sb_2_O, the rest of the CB is also predominately Ca d states with small amounts of Bi s, p and d, Ca s and p, and O p states.

Our calculated HSE06 + SOC electronic DoS curves are in agreement with previous calculations using the tight-binding linear-muffin-tin orbital method in the LDA and atomic-sphere approximations, where Bi/Sb p and O 2p were found to be the dominant contributors to the VB between 0 to −3.1 and −4.2 eV, respectively, and Ca 3d states were found to dominate the CB above the Fermi level.^36^ The two compounds have the same structure and are isoelectronic, resulting in similar total and partial DoS curves.

The electronic structures suggest predominantly ionic bonding in both compounds, although the small amount of mixing between states is suggestive of some covalent character. A key difference between the materials is the wider (more dispersive) VB in Ca_4_Bi_2_O, which indicates that the Bi p orbitals are better matched in energy with the unoccupied Ca states, allowing for better mixing than in the Sb analogue. The other main difference is in the position of Sb 5s and Bi 6s, which occur from −9 to −10 eV and −11 to −12 eV below the Fermi level, respectively, indicating that the latter have a higher binding energy.

**Fig. 3:**
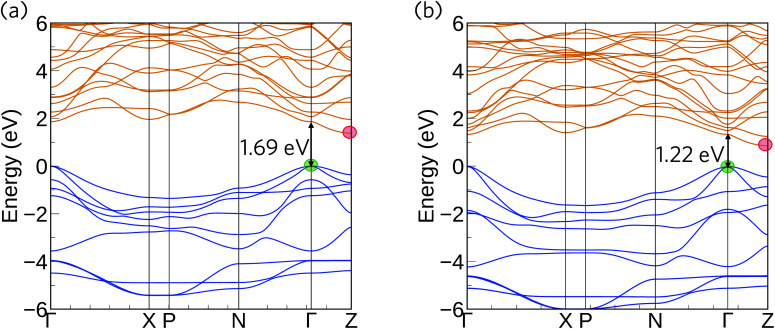
Calculated electronic band structures of Ca_4_Sb_2_O (a) and Ca_4_Bi_2_O (b) obtained at the HSE06 + SOC level of theory. The occupied valence bands are coloured blue and the unoccupied conduction bands are coloured yellow. The valence band maximum (VBM) is set to 0 eV. The VBM at *Γ* and the conduction band minima (CBM) at *Z* are marked with green and red circles respectively, and the size of the direct fundamental band gaps at *Γ* are marked by arrows.

The HSE06 + SOC calculated band structures for Ca_4_Sb_2_O and Ca_4_Bi_2_O are shown in Fig. 3. The CBM shows negligible SOC effects, as it is dominated by states from the lighter Ca atoms, whereas the correction gave rise to substantial upward shifts of around 0.18 and 0.55 eV in the VBM of Ca_4_Sb_2_O and Ca_4_Bi_2_O, respectively, due to the dominant contributions of states from the heavy Bi/Sb atoms. As a result, the inclusion of SOC results in a net band gap reduction in both compounds, illustrating the important role of relativistic effects on the electronic structure of these materials. Both compounds are indirect band gap semiconductors, with the VBM and CBM situated at the symmetry points *Γ* and *Z*, respectively. Ca_4_Sb_2_O has an indirect band gap of 1.38 eV, and a direct fundamental gap at *Γ* of 1.69 eV. Ca_4_Bi_2_O has an indirect band gap of 0.85 eV and a direct fundamental gap, again at *Γ*, of 1.22 eV. The smaller gap of Ca_4_Bi_2_O is due to the higher-lying Bi p states undergoing a stronger orbital interaction with Ca states, leading to a more dispersive VB and higher-energy VBM.

In both compounds, the VB and CB are very dispersive around the band edges, indicating high charge carrier mobilities. For Ca_4_Sb_2_O, the top of the valence band shows a larger dispersion along the *Γ*–*X* direction compared to the *Γ*–*N* and *Γ*–*Z* directions. This increased dispersion leads to a smaller hole effective mass of 0.269 *m*_e_ compared to the larger effective masses of 0.425 and 0.735 *m*_e_ along the other directions, as indicated in Table 3. The *Γ*–*X* direction is along the *c* axis in real space, and the lower effective masses can be attributed to the linear coordination around Ca and consequent improved mixing of the Ca d_*z*^2^_ with O and Sb states. The remaining Ca d states forming the CB are non-bonding and hence have a relatively flat dispersion compared to the VB, resulting in a comparatively larger electron effective mass of ∼0.949 *m*_e_ along the *Z*–*Γ* direction. These electron effective masses are still low, albeit larger than the hole effective masses.

**Table 3:** Hole and electron effective masses (*m*_0_) at the VBM and CBM of Ca_4_Sb_2_O and Ca_4_Bi_2_O, calculated at the HSE06 + SOC level of theory

System	VBM/*m*_e_	CBM/*m*_e_
Ca_4_Sb_2_O	0.269 (*Γ*–*X*) 0.425 (*Γ*–*N*) 0.735 (*Γ*–*Z*)	0.949 (*Z*–*Γ*)
Ca_4_Bi_2_O	0.168 (*Γ*–*X*) 0.268 (*Γ*–*N*) 0.558 (*Γ*–*Z*)	0.969 (*Z*–*Γ*)

The band structure of Ca_4_Bi_2_O exhibits similar characteristics to Ca_4_Sb_2_O around the CBM, with an electron effective mass of ∼0.969 *m*_e_ along the *Z*–*Γ* direction. The top of the VB, however, shows increased dispersion in all three directions relative to Ca_4_Sb_2_O, resulting in lighter hole effective masses of 0.168, 0.268 and 0.558 *m*_e_ along the *Γ*–*X*, *Γ*–*N* and *Γ*–*Z* directions, respectively. The CBM is dominated by Ca 3d states in both compounds, which makes the electron effective mass relatively insensitive to the metal anion. On the other hand, the VBM has large contributions from the Sb/Bi p states, and the lower hole effective masses suggest stronger orbital interactions in Ca_4_Bi_2_O. Provided the system is dopable, these low hole and electron effective masses would result in high carrier mobilities, suggesting the possibility of high electrical conductivity with both p- and n-type doping.

Unfortunately, we were unable to find any experimental data on the electronic structures of these compounds to compare our calculations to. While theoretical electronic structures and band gaps have been reported by Xia *et al.*,^36^ these are likely not a good reference point for comparison because of the methods used – in particular, the local density approximation (LDA) is known to severely underestimate band gaps.

**Fig. 4:**
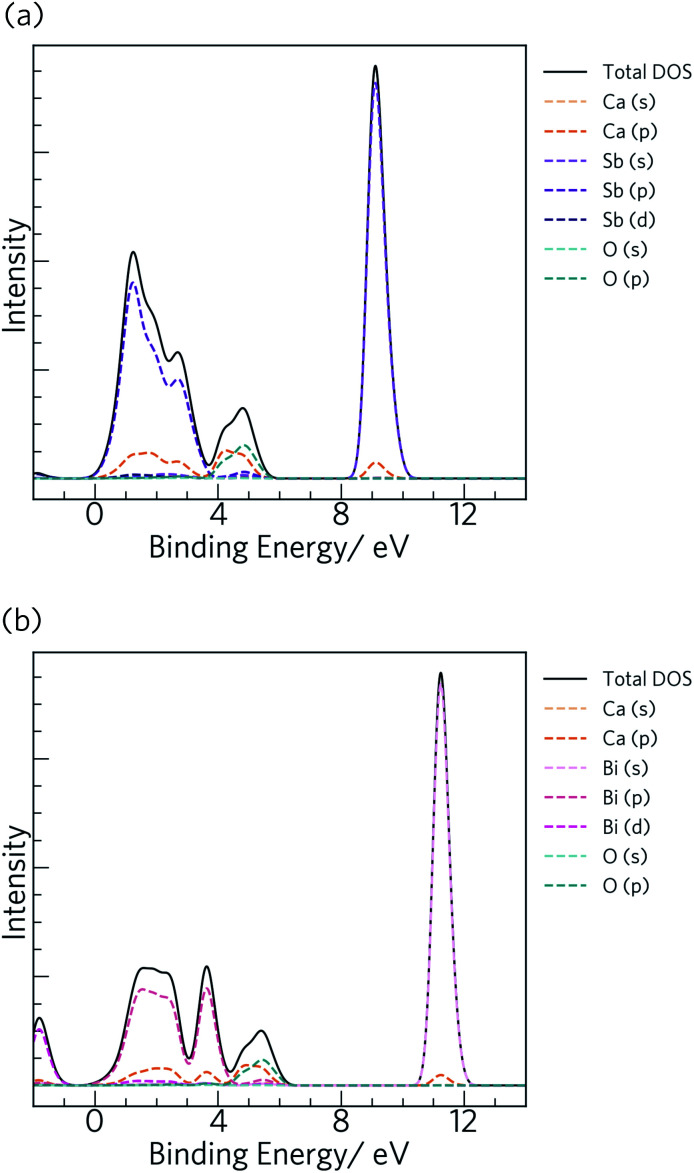
Simulated X-ray photoelectron spectra (0.3–2 keV) for Ca_4_Sb_2_O (a) and Ca_4_Bi_2_O (b), obtained from electronic density-of-states calculations performed at the HSE06 + SOC level of theory.

To provide a point of direct comparison to future experiments, we used our calculated total and partial density of states curves to simulate VB photoelectron spectra using the GALORE software package,^79^ which are shown in Fig. 4. The code computes the spectra by weighting the components of the atom-projected DoS according to their photoionization cross-sections,^80^ which depend on the probe radiation, orbital energies and orbital shapes. The calculated cross-sections do not however take into account the relativistic effects that would be exhibited by the heavy elements, which may result in a discrepancy between the simulated and measured spectra.^81^ Nonetheless, this method has proven to give good agreement with measured XPS spectra for many structures.^24,82–86^

### 3.2 Band alignment

**Fig. 5:**
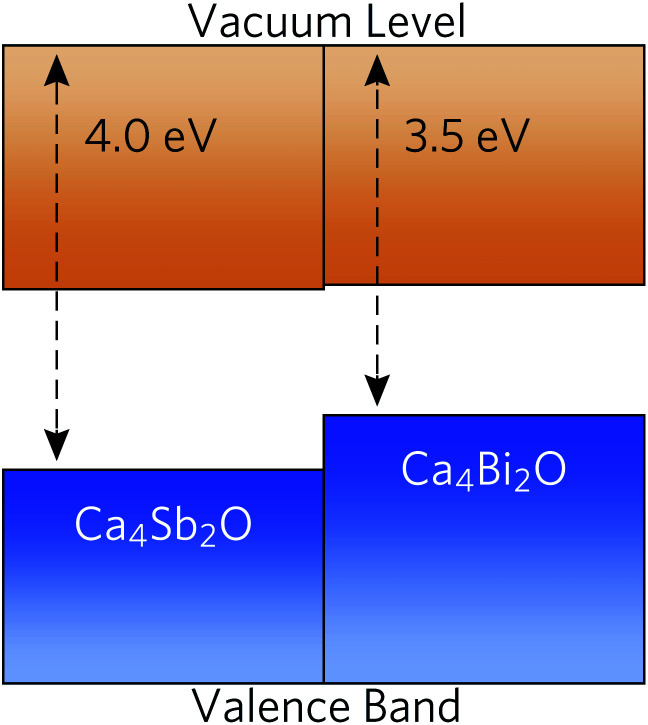
Band alignment of Ca_4_Sb_2_O and Ca_4_Bi_2_O using slab models, with energies calculated relative to the O 1s core levels. The valence and conduction bands are coloured in blue and orange respectively.

To predict whether the materials are more likely to be native p- or n-type semiconductors, we generated band-alignment diagrams using a slab model.^87^ The band alignment was performed using the core-level alignment approach,^88^ relative to the bulk O 1s core levels, at the HSE06 + SOC level of theory. The results are shown in Fig. 5.

Both compounds have high-lying valence-band maxima (VBM) relative to the vacuum level, which is a characteristic feature of native p-type semiconductors such as Si, Cu_2_O and ScN.^89–92^ The lower ionisation potential (lower-lying VBM) of Ca_4_Bi_2_O can be attributed to better overlap between the Bi p and Ca states and the resulting stronger orbital interactions and larger band dispersion. The high-lying VBM and conduction-band minima (CBM) in the two materials would result in low ionisation potentials and low electron affinities. Since many defect levels tend to lie within a certain absolute range of energies, band alignments also allow us to make an educated guess as to the p/n-type dopability of a material. Low ionisation potentials drive the formation of holes in semiconductors, and low electron affinites lead to limited n-type dopability due to charge compensation from the higher concentration of holes, resulting in a preference for p-type semiconducting behaviour.^93^ It is, however, important to conduct a full defect study in order to assess the position of the Fermi level.

### 3.3 Electronic transport properties

**Fig. 6:**
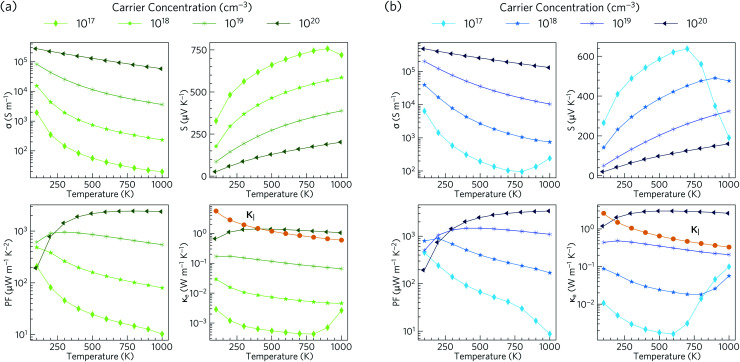
Calculated electronic transport properties as a function of temperature for p-type Ca_4_Sb_2_O (a) and Ca_4_Bi_2_O (b) at four different carrier concentrations: electrical conductivity (*σ*), Seebeck coefficient (*S*), power factor (PF) and electronic thermal conductivity (*κ*_e_). The lattice thermal conductivity (*κ*_l_; see Section 3.5) is indicated on the *κ*_e_ plots with orange lines for comparison.

Electronic transport properties for Ca_4_Sb_2_O and Ca_4_Bi_2_O obtained using our HSE06 + SOC electronic structures under p-type doping conditions, with carrier conentrations in the range of 10^17^ to 10^20^ cm^−3^, are presented in Fig. 6.

The electrical conductivity (*σ*) is given by *σ* = *neμ*, where *n* is the carrier concentration, *e* is the elementary charge and *μ* is the mobility. *σ* is therefore directly proportional to the concentrations of the charge carriers and their mobility. For Ca_4_Sb_2_O, the p-type electrical conductivities range from ∼20 S m^−1^ to ∼2.8 × 10^5^ S m^−1^. For Ca_4_Bi_2_O the electrical conductivities are larger and range from ∼95 to ∼4.9 × 10^5^ S m^−1^, which can be attributed to lower hole effective masses leading to higher mobilities. The room temperature electrical conductivities at a high hole concentration of 10^20^ cm^−3^ are 1.9 × 10^5^ and 3.5 × 10^5^ S m^−1^. These are larger than the value reported for polycrystalline Na_*x*_CoO_2−*δ*_ (5 × 10^4^ S m^−1^)^94^ and are comparable to Na_*x*_CoO_2−*δ*_ single crystals (5 × 10^5^ S m^−1^),^94,95^ which are among the highest reported electrical conductivities of oxide thermoelectrics.

**Fig. 7:**
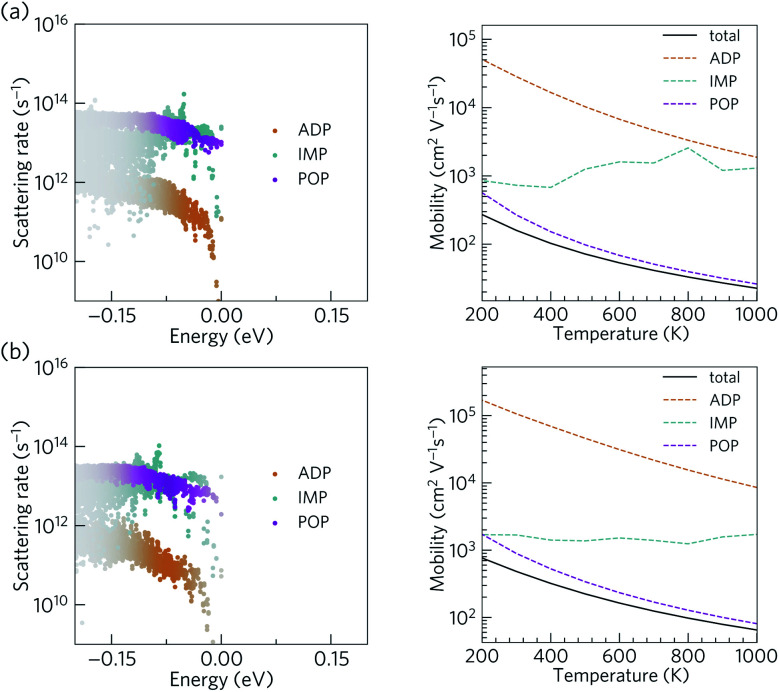
Calculated scattering rates and p-type mobilities of Ca_4_Sb_2_O (a) and Ca_4_Bi_2_O (b). The scattering rates are shown as a function of energy at a temperature of 300 K and a hole concentration of 10^19^ cm^−3^. The colour strength indicates the weighting from the energy derivative of the Fermi–Dirac distribution. The mobilities are shown as a function of temperature at a hole concentration of 10^19^ cm^−3^. Mobilities limited by polar optical phonon (POP), ionized impurity (IMP), and acoustic deformation potential (ADP) scattering processes are shown with magenta, cyan, and orange dashed lines, respectively, while the solid black line indicates the mobilities obtained by summing the rates of all three scattering mechanisms.

The scattering rates and mobilities are compared in Fig. 7 and indicate larger total mobilities in Ca_4_Bi_2_O than in Ca_4_Sb_2_O due to its lower m*. The hole mobilities in both Ca_4_Sb_2_O and Ca_4_Bi_2_O are found to be limited by polar optical phonon scattering, in agreement with the behaviour exhibited by other polar semiconductors.^48,96,97^ Polar optical scattering has also been identified as the dominant scattering mechanism in many of the current best-performing thermoelectrics including PbTe^98^ and SnSe.^99^ On the other hand, acoustic deformation scattering is found to have the least impact on the hole mobilities of both Ca_4_Sb_2_O and Ca_4_Bi_2_O. This can be attributed to the small absolute valence band edge deformation potentials of both materials (∼1.2 and ∼0.9 eV respectively).

The Seebeck coefficient (*S*) corresponds to the voltage induced in response to a temperature gradient, and can be either positive or negative depending on the dominant charge carrier type (+ve for holes and −ve for electrons). The p-type Seebeck coefficients for Ca_4_Sb_2_O range from about 26 μV K^−1^ at low *T* and high carrier concentration (10^20^ cm^−3^) to ∼757 μV K^−1^ at high temperature and low carrier concentration (10^17^ cm^−3^). A similar range of 20–638 μV K^−1^ is obtained for Ca_4_Bi_2_O.

The Seebeck coefficient depends on the electronic structure, and the difference could be related to the hole effective masses. In Ca_4_Sb_2_O, the larger hole inertial effective masses along the conduction direction would lead to higher band effective masses m^*^_band_ and, as a result, higher density of states effective masses m^*^_DoS_, due to the relationship:^100^

6 m^*^_DoS_ = *N*_V_^2/3^m^*^_band_

where *N*_V_ is the valley degeneracy (number of band extrema). This larger m^*^_DoS_ would then be responsible for the higher p-type Seebeck coefficients according to:7
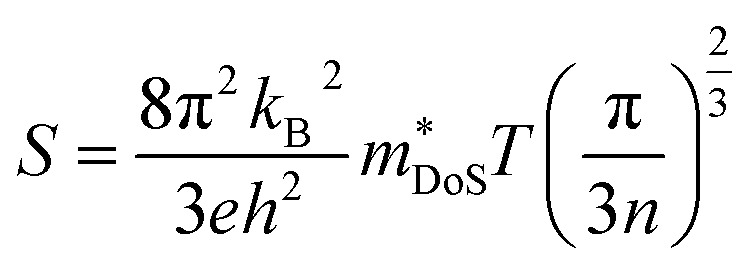
where, *k*_B_ is the Boltzmann constant, *e* is the elementary charge, *h* is Planck's constant, *n* is the carrier concentration and m^*^_DoS_ is the density of states effective mass.

The room-temperature Seebeck coefficients at low carrier concentrations of ≤10^19^ cm^−3^ for Ca_4_Sb_2_O and ≤10^18^ cm^−3^ for Ca_4_Bi_2_O are larger than the ∼176 μV K^−1^ at 300 K reported for dual-doped Zn_0.96_Al_0.02_Ga_0.02_O,^101^ which is reported to possess the highest *ZT* among n-type bulk oxide thermoelectrics. The maximum Seebeck coefficients of Ca_4_Sb_2_O and Ca_4_Bi_2_O are also higher than those reported for the majority of oxide thermoelectric materials.^95,101–106^

The power factors PF = *S*^2^*σ* are the numerator of the *ZT* expression and thus large PFs are essential for high thermoelectric performance. The combination of high *σ* and large *S* result in p-type PFs in the ranges of 10–2441 and 9–3389 μW m^−1^ K^−2^ for Ca_4_Sb_2_O and Ca_4_Bi_2_O respectively. These values are higher than the 810 μW m^−1^ K^−1^ reported for polycrystalline Na_*x*_CoO_2−*δ*_ at 800 K.^94^

The thermal conductivity, which appears in the denominator of the *ZT* equation, consists of two components: the electronic thermal conductivity *κ*_e_, which corresponds to heat conduction by charge carriers, and the lattice thermal conductivity *κ*_l_, which corresponds to heat transport through lattice vibrations (phonons). We show the *κ*_l_ values obtained from our calculations, (discussed in the following subsections) against the *κ*_e_ obtained from the electronic-structure calculations in Fig. 6. At carrier concentrations less than and equal to 10^19^ cm^−3^, the *κ*_e_ is smaller than the *κ*_l_ and the latter would therefore dominate the thermal transport, whereas at higher doping concentrations around 10^20^ cm^−3^ the electronic contribution becomes dominant above 400 and 170 K for Ca_4_Sb_2_O and Ca_4_Bi_2_O respectively.

### 3.4 Phonon dispersion and density of states

**Fig. 8:**
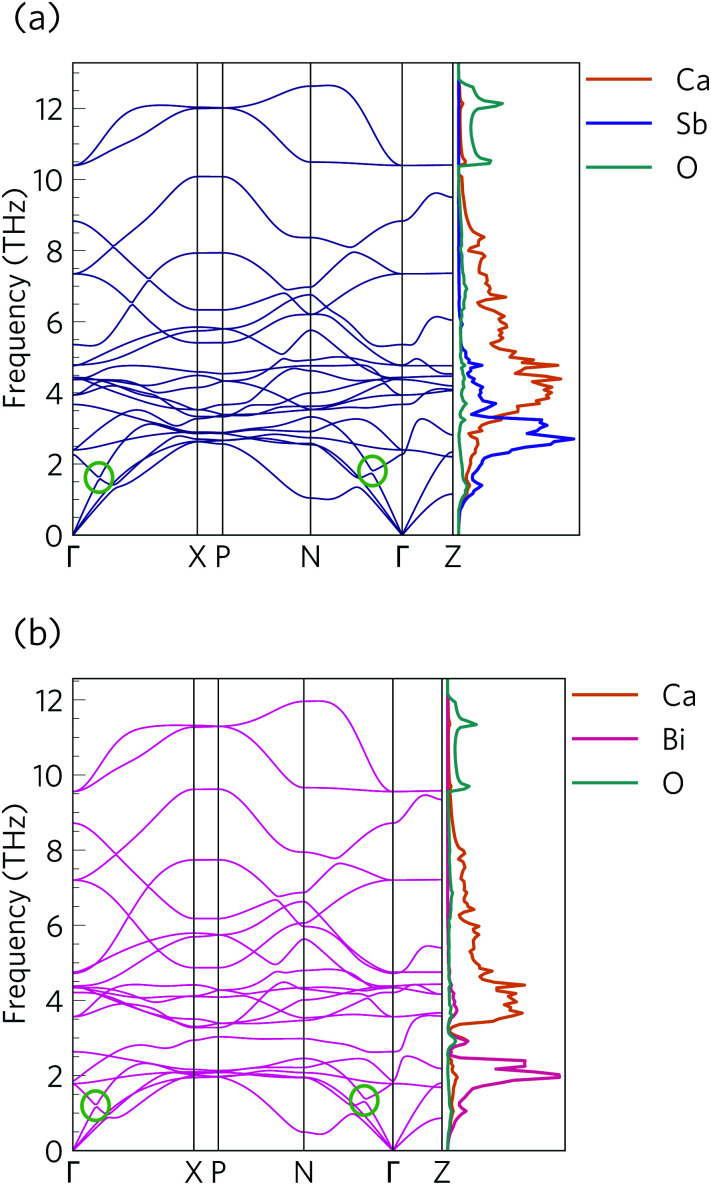
Harmonic phonon dispersion and atom-projected density of states (pDoS) curves for Ca_4_Sb_2_O (a) and Ca_4_Bi_2_O (b). Projections of the DoS onto Ca, Sb, Bi and O atoms are displayed in orange, purple, pink, and cyan, respectively. Avoided crossings between acoustic and optic modes are marked by green circles in the dispersions.

The phonon dispersions of Ca_4_Sb_2_O and Ca_4_Bi_2_O are shown together with the atom-projected phonon density of states (pDoS) curves in Fig. 8.

The primitive cells of both materials contain 7 atoms, resulting in 3*N* = 21 phonon bands at each wavevector. Both structures are dynamically stable, indicated by the absence of imaginary phonon modes in the dispersions.

Due to the inverse relationship between the reduced mass and frequency, the lower, mid and high-frequency phonon modes in both materials mostly comprise motions of the Sb/Bi, Ca and O atoms respectively. The Ca_4_Sb_2_O phonon spectrum is spread over a wider frequency range than that of Ca_4_Bi_2_O, which can be attributed to the heavier Bi atoms downshifting the frequencies.

The dispersion curves show relatively flat optic branches in the low-frequency region, which would result in low group velocities (*ν*_*λ*_). Both phonon dispersion curves also show avoided crossings between a low-lying optic mode and an acoustic mode along the *Γ*–*X* and *N*–*Γ* directions, which is a characteristic feature of “rattling” behaviour associated with heavy, weakly-bound atoms and suppressed thermal transport.^107–109^ Given that the low-frequency optic modes are dominated by Sb/Bi motion, we can infer that these atoms behave as rattlers. The large gap at the avoided crossing is indicative of strong hybridisation between the acoustic and optic modes, which modifies the dispersion and reduces the group velocity of the acoustic modes and, as these are the primary heat carriers, also reduces the thermal transport. Avoided crossings can also increase the phonon scattering rates and further suppress the heat transport. Due to the comparatively small unit cells, parts of the phonon spectrum are relatively sparse, which may limit the number of energy- and momentum-conserving scattering channels in these materials, in contrast to materials with large and complex unit cells such as Bi_2_Sn_2_O_7_.^24^ However, there is a relatively high density of optic modes in the low-frequency part of the spectrum, which may enable a large number of scattering channels for the heat-carrying acoustic modes.

### 3.5 Lattice thermal conductivity

**Fig. 9:**
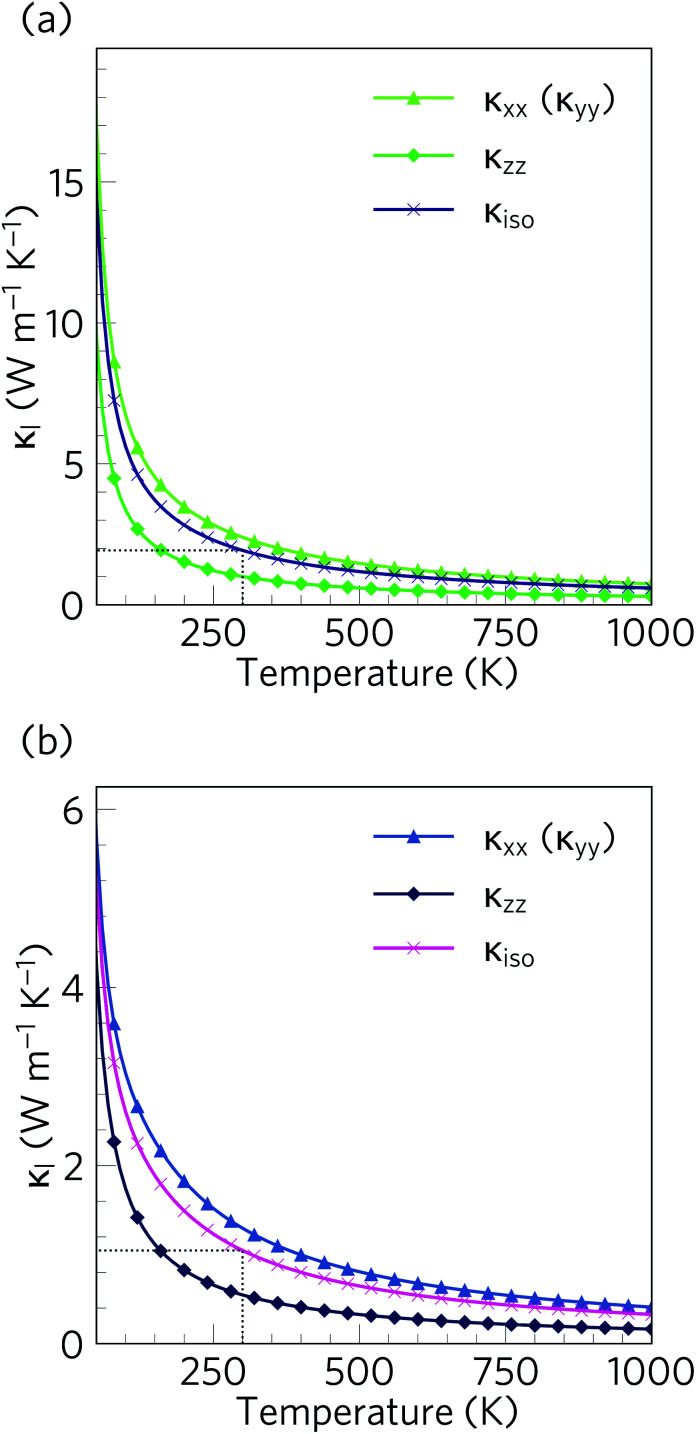
Calculated lattice thermal conductivity *κ*_l_ of Ca_4_Sb_2_O (a) and Ca_4_Bi_2_O (b) as a function of temperature. The principal *κ_xx_*, *κ_yy_*, and *κ_zz_* components of the *κ*_l_ tensor, corresponding to transport along the *a*, *b* and *c* axes respectively, are shown together with the diagonal average 
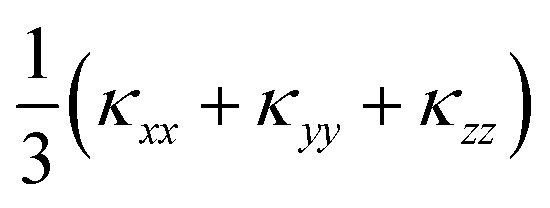
. On each plot the room-temperature (300 K) average *κ*_l_ is marked by a dotted black line.

The calculated lattice thermal conductivity *κ*_l_ of Ca_4_Sb_2_O and Ca_4_Bi_2_O as a function of temperature are compared in Fig. 9. As a result of the tetragonal symmetry of both structures, the thermal-conductivity tensors have two unique diagonal components, *viz. κ*_*xx*_/*κ*_*yy*_, corresponding to transport along the *a* and *b* axes, and *κ*_*zz*_, corresponding to transport along the *c* axis. The lower lattice thermal conductivity along the *c*-axis can be ascribed to weaker Ca–Sb/Ca–Bi chemical bonding (bond lengths ≈ 3.18 and 3.22 Å, respectively) compared to the stronger Ca–O bonds (≈2.34 and 2.36 Å) in the *ab* plane.

The *κ*_l_ of Ca_4_Sb_2_O is higher than that of Ca_4_Bi_2_O, with the room temperature average value of 1.93 W m^−1^ K^−1^ in the former being almost double the 1.05 W m^−1^ K^−1^ in the latter. The average *κ*_l_ approaches 0.79 and 0.45 W m^−1^ K^−1^ at 750 K, and decreases further to 0.60 and 0.33 W m^−1^ K^−1^ at 1000 K. The room temperature *κ*_l_ are comparable to the industry-standard PbTe,^110^ and are much lower than the majority of oxide thermoelectrics.^94,101,105,111^ At higher temperatures, the *κ*_l_ fall below 1 W m^−1^ K^−1^, and such “ultra-low” thermal conductivities are a feature of high-efficiency thermoelectrics. At 1000 K, the *κ*_l_ along the *c* direction falls to 0.16 W m^−1^ K^−1^, which is lower than the 0.20 W m^−1^ K^−1^ reported for SnSe along the layering direction.

The *κ*_l_ calculated using the relaxation-time approximation (RTA) only takes into account three-phonon scattering mechanisms, and does not account for other scattering processes such as electron–phonon and phonon-defect scattering. The model also neglects higher-order anharmonic terms, for example the fourth-order interactions, which are less restricted by the laws of energy and momentum conservation and can in some cases make a substantial contribution to the *κ*_l_. However, the RTA also does not account for the collective phonon excitations present in a full solution of the linearised Boltzmann transport equation (LBTE), and as such tends to underestimate the *κ*_l_,^112–115^ which partially compensates for the neglect of other scattering mechanisms and often gives good agreement with experiments. We also investigated the effect of isotope scattering and found only a very minor effect (Fig. S6†).

### 3.6 Modal contributions to the lattice thermal transport

**Fig. 10:**
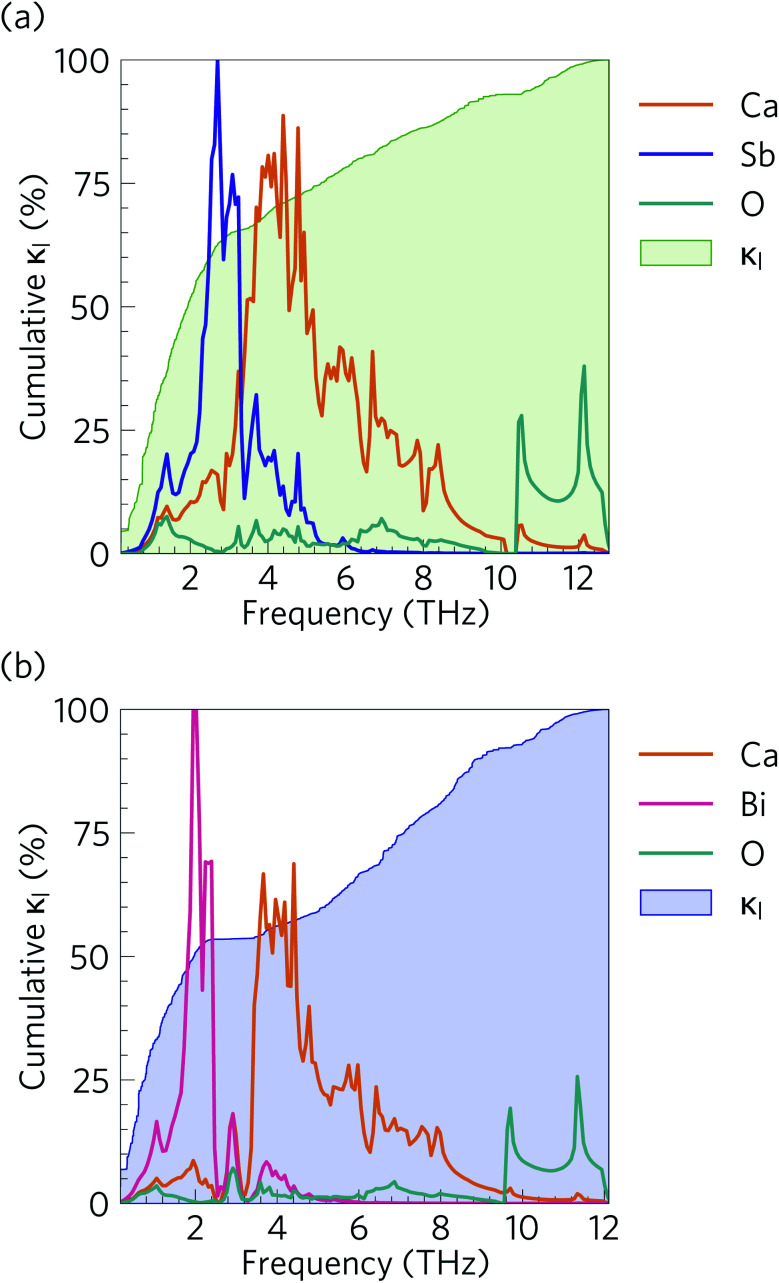
Cumulative % lattice thermal conductivity of (a) Ca_4_Sb_2_O and (b) Ca_4_Bi_2_O as a function of frequency at *T* = 300 K (green/blue shaded areas) overlaid against the phonon density of states projected onto Ca (orange), Sb (purple), Bi (pink) and O (cyan) atoms.

Fig. 10 compares the cumulative lattice thermal conductivity of Ca_4_Sb_2_O and Ca_4_Bi_2_O as a function of phonon frequency at 300 K. This analysis shows that the majority of the heat transport in both materials is through low-frequency phonon modes from 0–2.8 and 0–2.1 THz in Ca_4_Sb_2_O and Ca_4_Bi_2_O respectively. Comparing the accumulation with the phonon spectra in Fig. 8 shows that these upper limits correspond to the maximum frequencies of the acoustic modes along the *Γ*–*X* and *Γ*–*P* directions plus some low-lying optic modes. In Ca_4_Sb_2_O these modes make up 64% of the overall *κ*_l_, whereas the equivalent modes in Ca_4_Bi_2_O account for only ∼53% of the total lattice thermal conductivity. Analysing the cumulative *κ*_l_ along the *a*/*b* and *c* axes (Fig. S7†) produces a similar result. The majority of the heat transport in the two materials therefore occurs through acoustic and low-frequency optic modes.

**Fig. 11:**
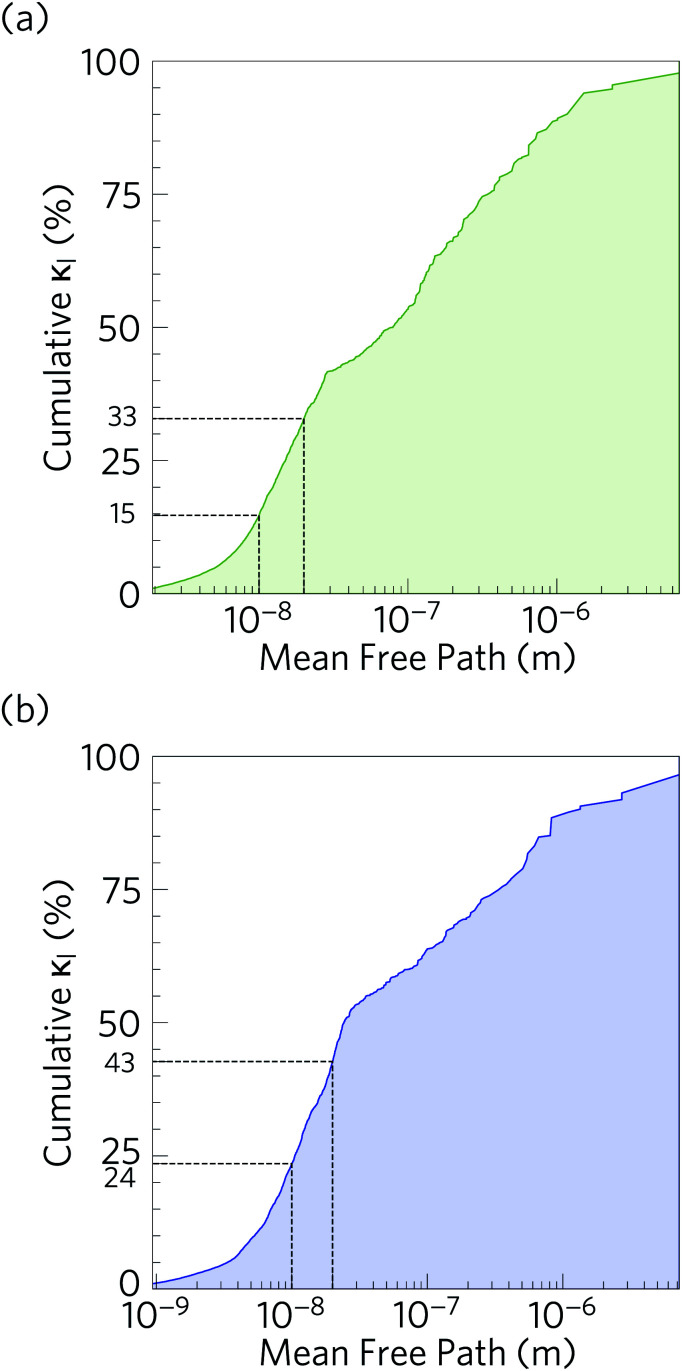
Cumulative % lattice thermal conductivity of Ca_4_Sb_2_O (a) and Ca_4_Bi_2_O (b) at *T* = 300 K as a function of the phonon mean free paths. The percentage of the lattice thermal conductivity from phonons with mean free paths less than 20 nm and 10 nm are indicated by dashed black lines.

It is also instructive to examine the cumulative *κ*_l_ as a function of the phonon mean free path *Λ*_*λ*_ = *ν*_*λ*_*τ*_*λ*_ (Fig. 11). In both structures, the *κ*_l_ increases to ∼10 μm, which would correspond to the longest mean free paths of the heat carrying modes in the two structures. We find that 85 and 76% of the thermal transport in Ca_4_Sb_2_O and Ca_4_Bi_2_O is through modes with mean free paths above 10 nm, while 67 and 57% is through modes with *Λ*_*λ*_ > 20 nm. This therefore indicates that the low lattice thermal conductivity of these materials could likely be further reduced by nanostructuring, a topic which we return to below.

**Fig. 12:**
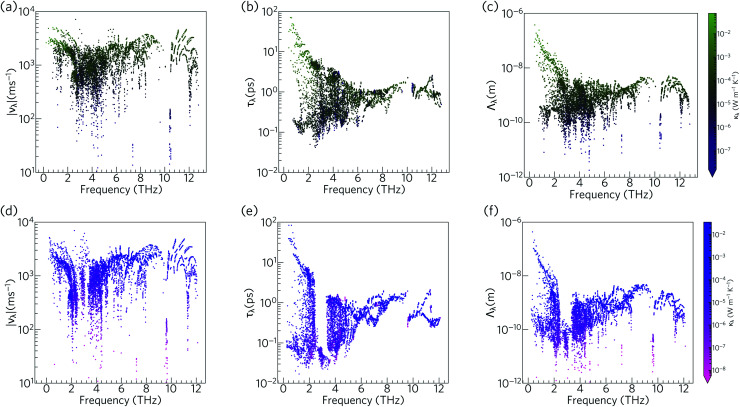
Analysis of the isotropically-averaged modal group velocity norms |*ν*_*λ*_| (a, d), lifetimes *τ*_*λ*_ (b, e), and mean-free paths *Λ*_*λ*_ (c, f) of Ca_4_Sb_2_O (a–c) and Ca_4_Bi_2_O (d–f) at *T* = 300 K. The data points are colour coded by the modal contributions to the *κ*_l_, *κ*_*λ*_, from purple to green (low to high *κ*_*λ*_) for Ca_4_Sb_2_O, and pink to blue (low to high *κ*_*λ*_) for Ca_4_Bi_2_O.

To further understand the range of modal contributions to the *κ*_l_, we plot the frequency spectra of the modal group velocity norms |*ν*_*λ*_|, lifetimes *τ*_*λ*_, and mean free path norms *Λ*_*λ*_ = |*ν*_*λ*_|*τ*_*λ*_ at 300 K (Fig. 12). We also investigated the directional anisotropy in the thermal transport by analysing separately the contributions of the modes making the dominant contributions to the transport along the *a*/*b* and *c* axes (Fig. S8†).

The group velocities of both Ca_4_Sb_2_O and Ca_4_Bi_2_O fall into a range of ∼10^1^ to 10^4^ m s^−1^. The fastest modes are seen around 1–2 THz, 6 THz, and 10–12 THz, reflecting the more dispersive phonon bands at these frequencies (*ν*_*λ*_ ≡ ∂*ω*_*λ*_/∂*q*). The overall spectra of the *ν*_*λ*_ is comparable to the hybrid perovskite (CH_3_NH_3_)PbI_3_ (MAPbI_3_), for which an ultralow lattice thermal conductivity of about 0.086 W m^−1^ K^−1^ has been predicted,^61^ and the generally low group velocities can be attributed to the presence of heavy elements in the structure and the consequent weak chemical bonding.

**Fig. 13:**
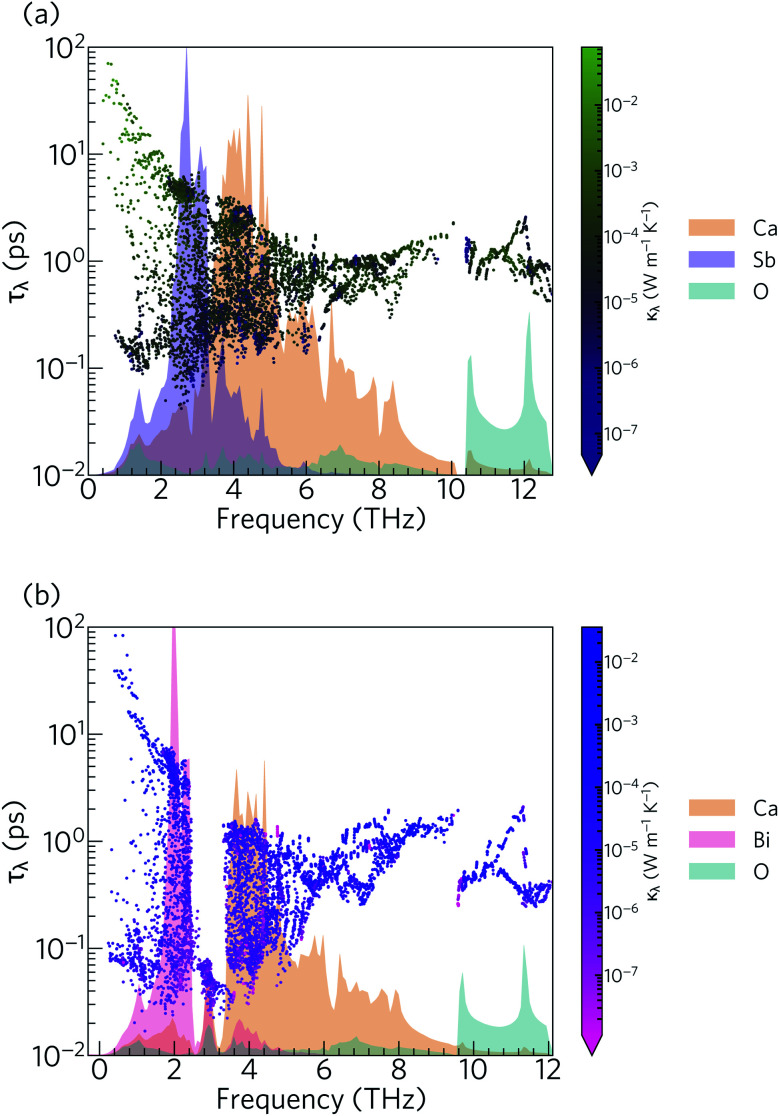
Frequency spectra of the phonon lifetimes *τ*_*λ*_ in Ca_4_Sb_2_O (a) and Ca_4_Bi_2_O (b) at *T* = 300 K, overlaid against the phonon density of states projected onto the Ca (orange), Sb (purple), Bi (pink) and O (cyan) atoms.

The phonon lifetimes in Ca_4_Sb_2_O span a range of 10^−1^ to 10^2^ ps, whereas for Ca_4_Bi_2_O a notable number of modes have *τ*_*λ*_ below 10^−1^ ps. Quantitatively, ∼17% of the modes in Ca_4_Bi_2_O have lifetimes smaller than 10^−1^ ps, compared to just 1.2% in Ca_4_Sb_2_O. Comparing the spectra of *τ*_*λ*_ with the phonon DoS (Fig. 13) shows that in Ca_4_Bi_2_O the modes with substantial Bi character have very short *τ*_*λ*_ compared to the Sb-based modes in Ca_4_Sb_2_O. In particular, there is a group of modes at around 3 THz in Ca_4_Bi_2_O for which the lifetimes are all below 0.1 ps, indicative of strong phonon scattering and large anharmonicity. The high-frequency O-based modes also have shorter lifetimes in Ca_4_Bi_2_O than in Ca_4_Sb_2_O, whereas the lifetimes of the mid-frequency Ca-based modes are quite similar in both materials.

The analysis in Fig. 12 shows that the lower-frequency modes generally have faster group velocities and longer lifetimes, and hence longer mean-free paths. This is consistent with the accumulations of the *κ*_l_ with respect to frequency and mean-free path (Fig. 10 and 11), which show that these modes contribute most to the thermal transport. The similar spread of group velocities but shorter mode lifetimes in Ca_4_Bi_2_O compared to Ca_4_Sb_2_O result in 73% of the modes having mean free paths less than 1 nm, as opposed to 55% in Ca_4_Sb_2_O. The majority of the *Λ*_*λ*_ are therefore less than 10 nm in both structures. The longest *Λ*_*λ*_ in Ca_4_Sb_2_O are 132 nm and 1.06 μm for transport in the *ab* plane and along the *c*-axis, respectively, while for Ca_4_Bi_2_O the maximum mean-free paths are 125 nm and 1.15 μm along the two directions. Although the longest *Λ*_*λ*_ for transport along the *c* axis is larger in Ca_4_Bi_2_O than in Ca_4_Sb_2_O, the average is shorter, at 902 pm compared to 1.07 nm.

Overall, the phonon modes in Ca_4_Sb_2_O and Ca_4_Bi_2_O are characterised by low group velocities and short phonon lifetimes, leading to short mean-free paths. While the group velocities in both materials span a similar range, the lower density of long-lived modes in Ca_4_Bi_2_O results in shorter average mean-free paths and thus a lower *κ*_l_.

### 3.7 Microscopic origin of short phonon mode lifetimes

The analysis in the previous section indicates that the phonon lifetimes in Ca_4_Sb_2_O and Ca_4_Bi_2_O are unusually short and are also the main origin of the lower *κ*_l_ of Ca_4_Bi_2_O. To understand the origin of the short lifetimes, and of the difference between the two materials, we performed the analysis outlined in ref. 59.

The *τ*_*λ*_ are calculated from the phonon linewidths *Γ*_*λ*_ from the relationship *τ*_*λ*_ = 1/2*Γ*_*λ*_. The *Γ*_*λ*_ are obtained as the imaginary part of the phonon self-energy by summing over contributions from energy- and (crystal) momentum-conserving three-phonon interactions between the reference mode *λ* and pairs of interacting modes *λ*′ and *λ*′′. The contribution of each scattering event to the overall linewidth depends on the three-phonon interaction strengths *Φ*_*λλ*′*λ*′′_, which describe the physical coupling between modes and depends on the third-order force constants, and a term that captures the temperature dependence of the linewidth *via* changes in the mode occupation numbers *n*_*λ*_.

An approximate *Γ*_*λ*_ can be written as the product of an averaged phonon interaction strength *P*_*λ*_ and a two-phonon weighted joint density of states *N*_2_(*q*, *ω*) (w-JDoS):^59^8
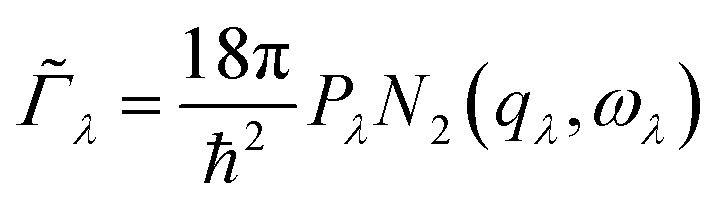


The *P*_*λ*_ are the averaged phonon–phonon interaction strengths for each mode calculated as:9
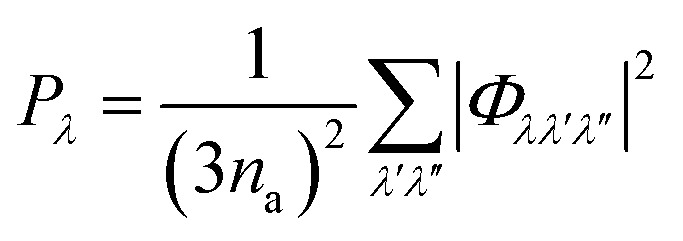
where *n*_a_ is the number of atoms in the primitive cell, and 3*n*_a_ is thus the number of bands at each phonon wavevector *q*.

*N*_2_(*q*, *ω*) counts the number of energy-conserving scattering pathways available to a mode with wavevector *q* and frequency *ω*, and is the sum of two separate functions corresponding to collision (Type 1) and decay (Type 2) processes:

10 *N*_2_(*q*,*ω*) = *N*^(1)^_2_(*q*,*ω*) + *N*^(2)^_2_(*q*,*ω*)

11
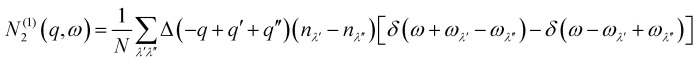
12
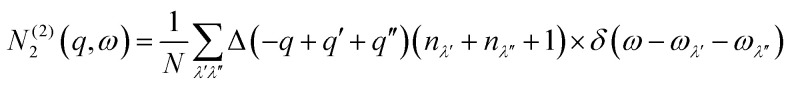
where the functions *δ* and Δ enforce conservation of energy and momentum respectively. For comparing between materials it is convenient to average over phonon wavevectors to obtain functions of frequency only, *i.e.*:

13 *N̄*_2_(*ω*) = *N̄*^(1)^_2_(*ω*) + *N̄*^(2)^_2_(*ω*)

14
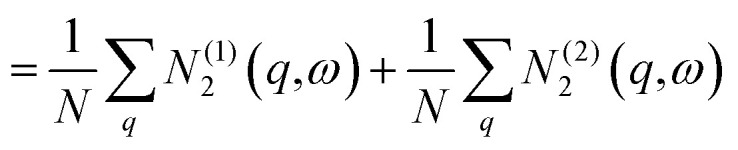


**Fig. 14:**
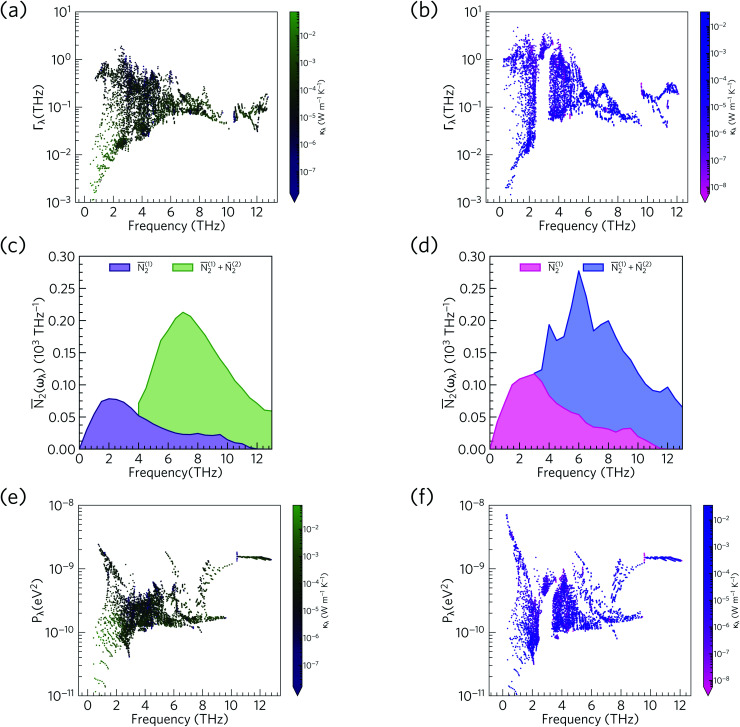
Comparison of the phonon linewidths *Γ*_*λ*_ (a/b), weighted two-phonon density of states *N̄*_2_(*ω*) (c/d) and averaged phonon–phonon interaction strengths *P*_*λ*_ (e/f) in Ca_4_Sb_2_O (left-hand panel) and Ca_4_Bi_2_O (right-hand panel). The *Γ*_*λ*_ and *N̄*_2_(*ω*) are shown at *T* = 300 K and *N̄*_2_(*ω*) are shown separately for collision (*N̄*^(1)^_2_) and decay processes (*N̄*^(2)^_2_). As in Fig. 12, the data points are colour coded by *κ*_l_ from purple to green (small to large *κ*_l_) for Ca_4_Sb_2_O and from pink to blue (small to large *κ*_l_) for Ca_4_Bi_2_O. Note that the *y* axes in subplots a/b and e/f are on a logarithmic scale, whereas subplots c/d are on a linear scale.

Fig. 14 compares the mode linewidths of Ca_4_Sb_2_O and Ca_4_Bi_2_O at 300 K with the averaged three-phonon interaction strengths *P*_*λ*_ and the w-JDoS functions *N̄*_2_(*ω*). For both compounds, the low-frequency modes show narrower linewidths (longer lifetimes) than the high-frequency modes, and the shorter lifetimes of Ca_4_Bi_2_O are reflected in the broader linewidths compared to Ca_4_Sb_2_O. Notably, Ca_4_Bi_2_O has a group of modes with broad linewidths at around 3 THz, which coincides with the very short lifetimes in this region of the spectrum (*c.f.*Fig. 12).

**Fig. 15:**
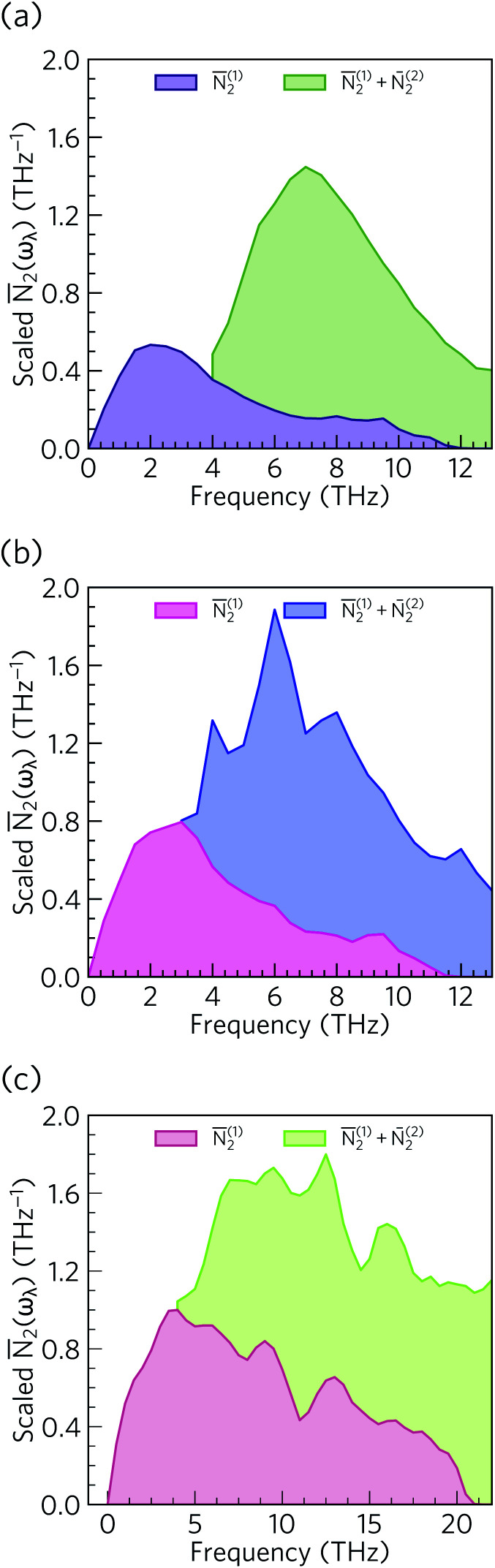
Comparison of the *N̄*_2_(ω) of Ca_4_Sb_2_O (a), Ca_4_Bi_2_O (b) and α-Bi_2_Sn_2_O_7_^24^ (c). Each function is scaled by a factor of 1/3*n*_a_^2^ to account for the larger primitive cell of α-Bi_2_Sn_2_O_7_.

From the *N*_2_(*ω*), it can be seen that the at low frequencies collision events dominate the phonon scattering, whereas at high frequencies decay processes dominate, and a combination of the two are active at intermediate frequencies. Comparing the *N̄*_2_(ω) of Ca_4_Sb_2_O and Ca_4_Bi_2_O to α-Bi_2_Sn_2_O_7_ ^24^ (Fig. 15) and scaling by 1/3*n*_a_^2^ to account for the different sizes of the respective primitive cells, shows that there are a comparable number of scattering pathways for low- and medium-frequency modes in Ca_4_Bi_2_O, but not Ca_4_Sb_2_O. The *N*_2_(*ω*) is determined purely by the phonon spectrum, and a high density of energy- and momentum-conserving scattering pathways is generally favoured by a dense phonon spectrum with a narrow band dispersion. In α-Bi_2_Sn_2_O_7_, the large and complex unit cell and weak chemical bonding produces a large number of scattering channels across the frequency spectrum. In Ca_4_Bi_2_O, the *N̄*_2_(*ω*) is comparable to that of Bi_2_Sn_2_O_7_ at low frequencies because of the significant overlap between the acoustic and low-frequency optic modes, and the avoided crossing between the acoustic and optic modes visible in the dispersion curves (*c.f.*Fig. 8) is also expected to play a role in increasing the acoustic-mode scattering phase space. This difference can be attributed to the fact that the low-frequency optic modes are dominated by motion of the heavier Sb/Bi atoms, and therefore occur at lower frequencies in Ca_4_Bi_2_O. In contrast to α-Bi_2_Sn_2_O_7_, the phonon dispersions of Ca_4_Sb_2_O and Ca_4_Bi_2_O are less dense and more dispersive at high frequencies, which results in the high-frequency modes having longer lifetimes. However, this does not have a significant impact on the thermal transport, as the majority of the heat transport is through the acoustic and the low-frequency optic modes (*c.f.*Fig. 10).

The w-JDoS functions do not completely explain the frequency dependence of the mode linewidths, as the modes also depend on the phonon interaction strengths *P*_*λ*_ as per eqn (8). The averaged phonon–phonon interaction strengths in Ca_4_Sb_2_O and Ca_4_Bi_2_O are comparable to the GaAs and CdTe^61^ and are considerably stronger than in α-Bi_2_Sn_2_O_7_.^24^ In Ca_4_Sb_2_O, the interactions generally span a range of 10^−10^ to 10^−9^ eV^2^, with notable spikes between 1 and 3 THz, at ∼6 Thz and above 8 THz. In Ca_4_Bi_2_O, the *P*_*λ*_ for some of the low-frequency modes are an order of magnitude stronger than in Ca_4_Sb_2_O. Comparison of the *Γ*_*λ*_, *N̄*_2_(*ω*) and *P*_*λ*_ shows that at low frequencies the strong physical coupling between modes compensates for the relatively low number of scattering pathways in the low-frequency region to produce broad linewidths. We therefore conclude that intrinsic anharmonicity is a key contributor to the short mode lifetimes in Ca_4_Sb_2_O and Ca_4_Bi_2_O. We attribute the stronger low-frequency phonon–phonon interaction strengths in Ca_4_Bi_2_O to the larger and more polarisable Bi anion giving rise to larger third-order force constants and stronger phonon–phonon interaction strengths.

Overall, from this analysis we can attribute the short phonon lifetimes and low *κ*_l_ of Ca_4_Sb_2_O and Ca_4_Bi_2_O to strong acoustic-mode scattering: (1) due to strong anharmonic phonon–phonon interactions; and (2) to a high density of energy and momentum-conserving scattering pathways produced by overlap between the acoustic and low-frequency optic modes. Both effects are enhanced in Ca_4_Bi_2_O compared to Ca_4_Sb_2_O, resulting in the latter having a ∼50% smaller *κ*_l_.

### 3.8 Nanostructuring

Alloying and nanostructuring are both widely-employed strategies to lower the *κ*_l_ and enhance the *ZT* of thermoelectric materials. Whereas alloying can often be detrimental to the electrical conductivity by decreasing carrier mobility,^21^ provided the length scale is designed to only scatter phonons the ‘bottom-up’ nanostructuring approach can decrease the *κ*_l_ while largely preserving the electronic transport properties. Grain-boundary scattering in particular is a useful strategy for selectively scattering fast, long-wavelength acoustic phonon modes.^62,116^

**Fig. 16:**
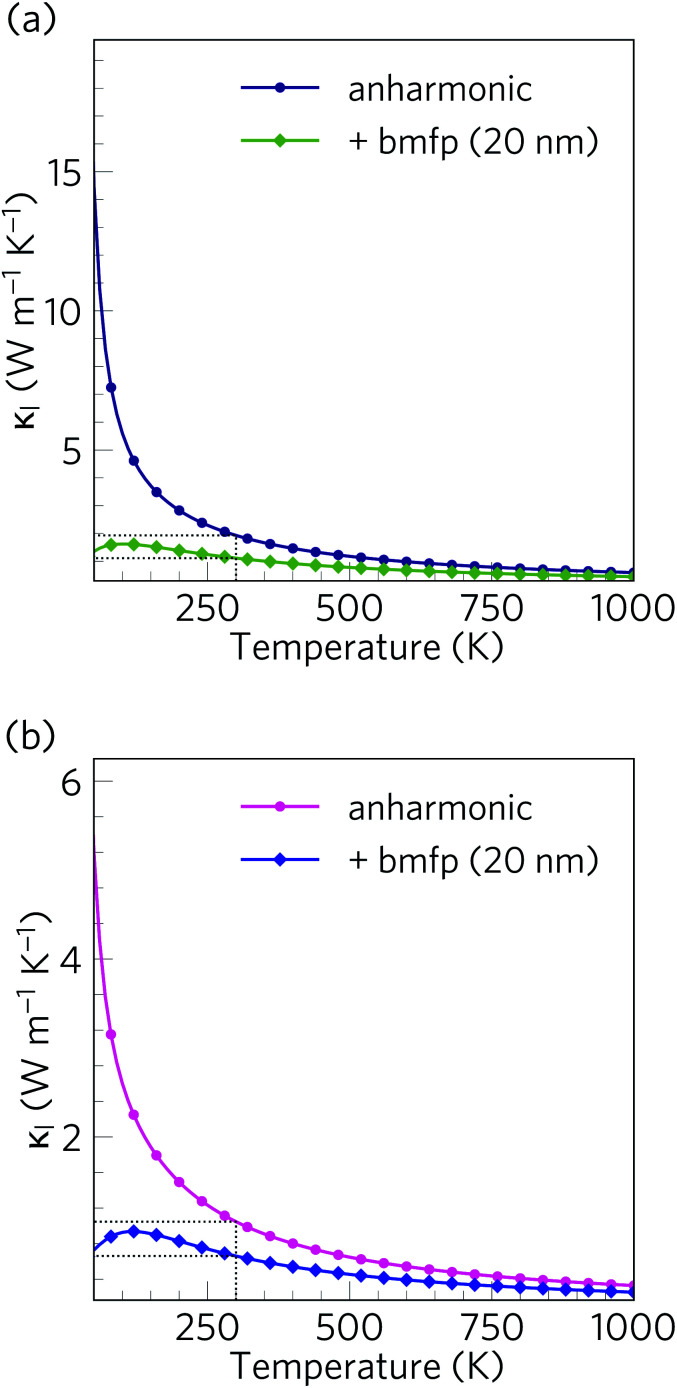
Isotropically averaged lattice thermal conductivity *κ*_l_ of Ca_4_Sb_2_O (a) and Ca_4_Bi_2_O (b) as a function of temperature without (circles) and with (diamonds) a boundary-scattering model applied to mimic a crystal grain size of 20 nm.

We investigated the effect of nanostructuring on the *κ*_l_ in Ca_4_Sb_2_O and Ca_4_Bi_2_O using the simple boundary-scattering model implemented in the Phono3py code, which limits the mean-free paths of modes which have |*Λ*_*λ*_| larger than a cutoff value. We tested a grain size of 20 nm to coincide with the level of nanostructuring attained by Hsu *et al.* with AgPb_18_SbTe_20_.^117^ As shown in Fig. 16, this decreases the room temperature average *κ*_l_ of Ca_4_Sb_2_O and Ca_4_Bi_2_O by around 42% and 37% to 1.11 and 0.66 W m^−1^ K^−1^, respectively. This is a considerable reduction, and indicates that nanostructuring can be efficiently used to further increase the thermal resistance in both materials.

### 3.9 Thermoelectric figure of merit

**Fig. 17:**
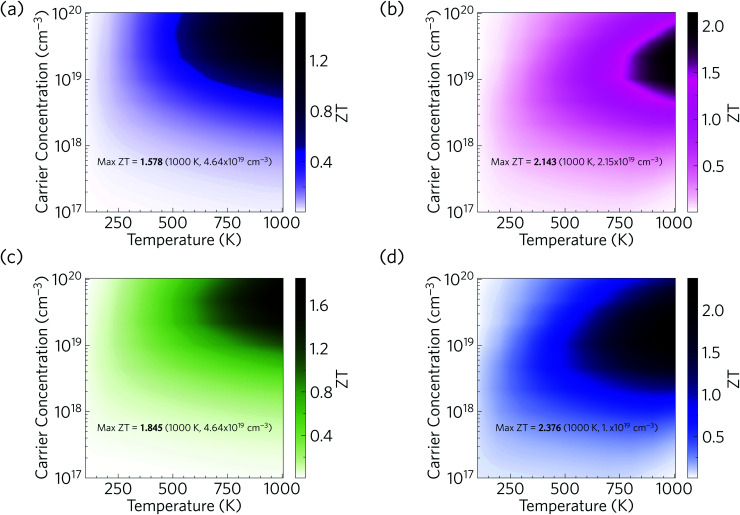
Predicted thermoelectric figure of merit *ZT* of p-type doped Ca_4_Sb_2_O (a/c) and Ca_4_Bi_2_O (b/d) as a function of temperature and carrier concentration. (a) and (b) show predictions based on the lattice thermal conductivity of bulk single crystals, while (c) and (d) show the enhanced *ZT* obtained by nanostructuring down to a grain size of 20 nm to limit the *κ*_l_.

We now combine our calculated electrical properties and lattice thermal conductivity to predict the thermoelectric figure of merit *ZT* for Ca_4_Sb_2_O and Ca_4_Bi_2_O (Fig. 17). We predict a maximum p-type *ZT* of 1.58 for Ca_4_Sb_2_O at *T* = 1000 K and a carrier concentration of 4.64 × 10^19^ cm^−3^, and 2.14 for Ca_4_Bi_2_O at *T* = 1000 K and a carrier concentration of 2.15 × 10^19^ cm^−3^. At room temperature (300 K), we predict a maximum *ZT* of 0.16 and 0.31 for Ca_4_Sb_2_O and Ca_4_Bi_2_O respectively, at carrier concentrations of 2.15 × 10^19^ and 4.64 × 10^18^ cm^−3^. The room-temperature *κ*_l_ of 1.93 and 1.05 W m^−1^ K^−1^ are substantially reduced to 0.60 and 0.33 W m^−1^ K^−1^ at 1000 K, which, if *κ*_e_ remains negligible, would produce a ∼6× increase in the *ZT* with the additional 3× increase coming from the increase in temperature. The remainder of the enhancement comes from an improvement in the electrical properties at elevated temperatures. As noted earlier, Ca_4_Bi_2_O has slightly higher PFs than Ca_4_Sb_2_O, which along with its lower *κ*_l_ (∼2 times lower) are responsible for its higher *ZT*.

Comparing the predicted *ZT* with other oxide thermoelectrics indicates that both materials may have comparable or slightly larger room temperature figures of merit than the majority of existing p-type oxide thermoelectrics and potentially larger *ZT* at elevated temperatures. Single crystals of Na_*x*_CoO_2−*δ*_, one of the earliest p-type oxide thermoelectrics discovered, have been reported to show a *ZT* of 0.03 at 300 K and 1.2 at 800 K.^94^ Single crystals of Ca_3_Co_4_O_9_ have been reported to have a *ZT* of ∼0.07 at 300 K and 0.87 at 973 K.^102^ Bi_2_Sr_2_Co_2_O_*y*_, another p-type oxide thermoelectric, was reported to have a *ZT* of less than 0.1 at room temperature and about 1.1 at 973 K.^104^ The main reason for the higher *ZT* values of Ca_4_Sb_2_O and Ca_4_Bi_2_O is their low lattice thermal conductivity. Our predicted values of <2 W m^−1^ K^−1^ at room temperature and <1 W m^−1^ K^−1^ at higher *T* are considerably smaller than the 19 and 5 W m^−1^ K^−1^ of single-crystal Na_*x*_CoO_2−*δ*_ at 300/800 K,^94^ the 3/2 W m^−1^ K^−1^ of Ca_3_Co_4_O_9_ measured at 300 K and higher temperature,^102^ and the >2 W m^−1^ K^−1^ of Bi_2_Sr_2_Co_2_O_*y*_ even at elevated temperature.^104^

Our predictions therefore suggest that, provided they can be doped, Ca_4_Sb_2_O and Ca_4_Bi_2_O could potentially serve as very promising p-type thermoelectric materials. We also note that there is also potential to further enhance the *ZT* by nanostructuring to further reduce the *κ*_l_. As depicted in Fig. 17, nanostructuring to 20 nm is predicted to further increase the maximum *ZT* of Ca_4_Sb_2_O and Ca_4_Bi_2_O to 1.85 and 2.38 respectively, which amount to modest increases of 17 and 11% over the bulk values.

## 4 Conclusions

In summary, we have performed a detailed investigation of the thermoelectric properties of two mixed-anion oxide systems, Ca_4_Sb_2_O and Ca_4_Bi_2_O.

Both Ca_4_Sb_2_O and Ca_4_Bi_2_O possess good electronic transport properties, comparable to other known oxide thermoelectric materials. Despite the predominantly ionic bonding, the inhomogenous chemical bonding and heavy metals produce a combination of low phonon group velocities and short phonon lifetimes. These together result in room-temperature lattice thermal conductivities of 1.93 and 1.05 W m^−1^ K^−1^, which are considerably lower than most other oxide thermoelectrics. Analysis of the *κ*_l_ shows that the majority of the heat transport occurs through acoustic and low-frequency optic modes for which the mean-free paths are heavily limited by suppressed phonon lifetimes. The heavy metals in the mixed-anion structures produce relatively flat, low-frequency optic bands that modify the dispersion by avoided crossings with the acoustic modes and also provide a high density of phonon scattering channels for the heat-carrying modes. These effects are further enhanced by strong physical coupling between modes, indicative of large phonon anharmonicity. The heavier and more polarisable Bi anions in Ca_4_Bi_2_O result in stronger phonon–phonon interactions than in Ca_4_Sb_2_O and an approx. two-fold reduction in the *κ*_l_.

We predict high p-type thermoelectric figures of merit approaching ∼1–2 at high temperatures, which can be attributed both to the good electronic transport properties and to the low lattice thermal conductivity. Our calculations also highlight the possibility of further lowering the thermal conductivity and enhancing the *ZT* by nanostructuring.

Despite there being no upper limit to *ZT*, most thermoelectric materials, including some of the industry standards, attain a maximum *ZT* of ∼1 at the operating temperature. Breaking this threshold is challenging due to the interdependence of the electrical properties *S*, *σ* and *κ*_e_, and to the difficulty of finding materials with intrinsically low *κ*_l_. The latter requirement has historically led to materials made from heavy elements such as Pb and Te which are, unfortunately, often also rare and/or toxic. The mixed-anion systems investigated in this study consist of earth-abundant and non-toxic elements with the added advantages of high chemical and thermal stability, and have the potential to reach a *ZT* above 1 at elevated temperature. This suggests they are viable candidates for large-scale applications, and thus warrant further investigation. Future theoretical work should extend this study to establish the defect chemistry of the two materials to confirm whether they are dopable and to identify suitable dopants.

Provided this is the case, mixed-anion systems such as Ca_4_Sb_2_O and Ca_4_Bi_2_O could constitute a new class of high-performance oxide thermoelectrics and provide an interesting new direction for future research.

## Conflicts of interest

The authors declare no competing financial interests.

## Supplementary Material

TA-009-D1TA03649A-s001
