# Supplementary material for: Ca4Sb2O and Ca4Bi2O: two promising mixed-anion thermoelectrics
Source: J Mater Chem A Mater. 2021 Aug 2;9(36):20417–35. doi: 10.1039/d1ta03649a (PMC8454491; doi:10.1039/d1ta03649a)
Supplement: TA-009-D1TA03649A-s001 [file TA-009-D1TA03649A-s001.pdf]

Electronic Supplementary Material (ESI) for Journal of Materials Chemistry A.
This journal is © The Royal Society of Chemistry 2021

Ca_4Sb_2O and Ca_4Bi_2O: Two Promising Mixed-Anion Thermoelectrics — Supplementary Information

Warda Rahim,[†,‡] Jonathan M. Skelton,[¶] and David O. Scanlon[*,†,‡,§]

†*Department of Chemistry, University College London, 20 Gordon Street, London WC1H 0AJ, UK*

‡*Thomas Young Centre, University College London, Gower Street, London WC1E 6BT, UK*

¶*Department of Chemistry, University of Manchester, Oxford Road, Manchester M13 9PL, UK*

§*Diamond Light Source Ltd., Diamond House, Harwell Science and Innovation Campus, Didcot, Oxfordshire OX11 0DE, UK*

E-mail: d.scanlon@ucl.ac.uk

Convergence of the Total Energy with Respect to Plane-Wave Cutoff and *k*-Point Sampling

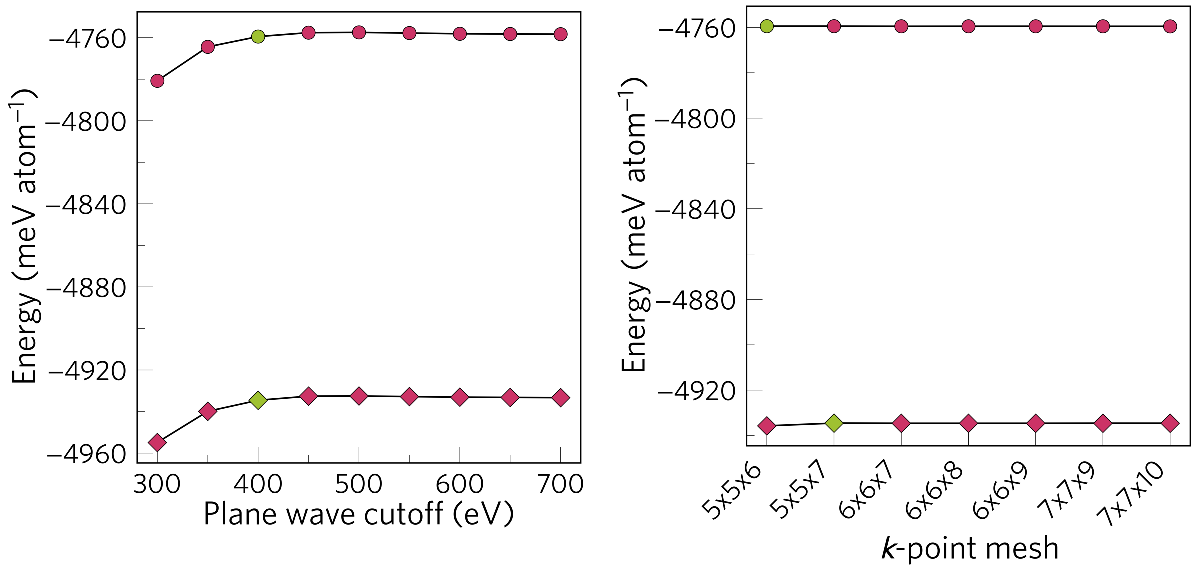

(a) **(b)**

Figure S1: Calculated total energy of Ca_4Sb_2O (diamonds) and Ca_4Bi_2O (circles) as a function of the plane wave kinetic-energy cutoff (a) and the k-point sampling mesh (b). The cutoff and k-point mesh converged to < 5 meV per atom and 1 meV per atom respectively are highlighted in green.

Convergence of the Electronic Transport Properties with Respect to the Interpolation Factor

The interpolation factor in the AMSET package[1] controls the density of k-points in the interpolated band structures, with the number of interpolated k-points being approximately equal to the interpolation factor times the number of k-points in the DFT calculation. The transport properties can be highly sensitive to the k-point density, so it is important to explicitly converge the calculated results with respect to this parameter. Figure S2 and Figure S3 illustrate the testing performed to select an appropriate interpolation factor for the calculations performed in this work. The k-point sampling mesh used for the DFT calculation was $14\times14\times18$. The transport properties are converged with an interpolated k-point mesh of $65\times65\times89$ but for greater accuracy, we used $69\times69\times93$ for both materials.

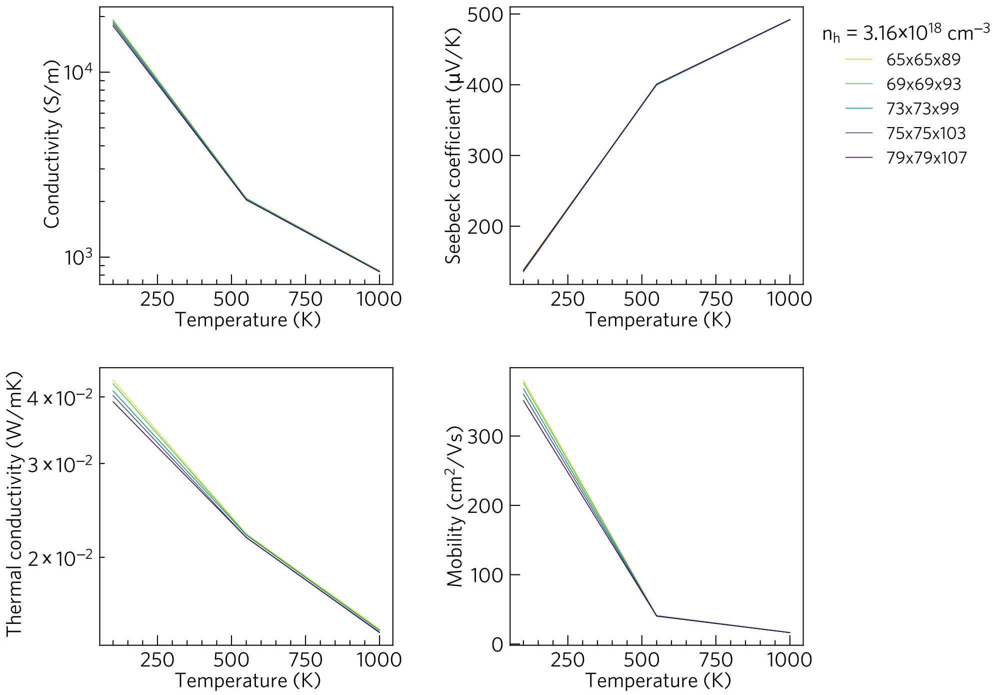

(a)

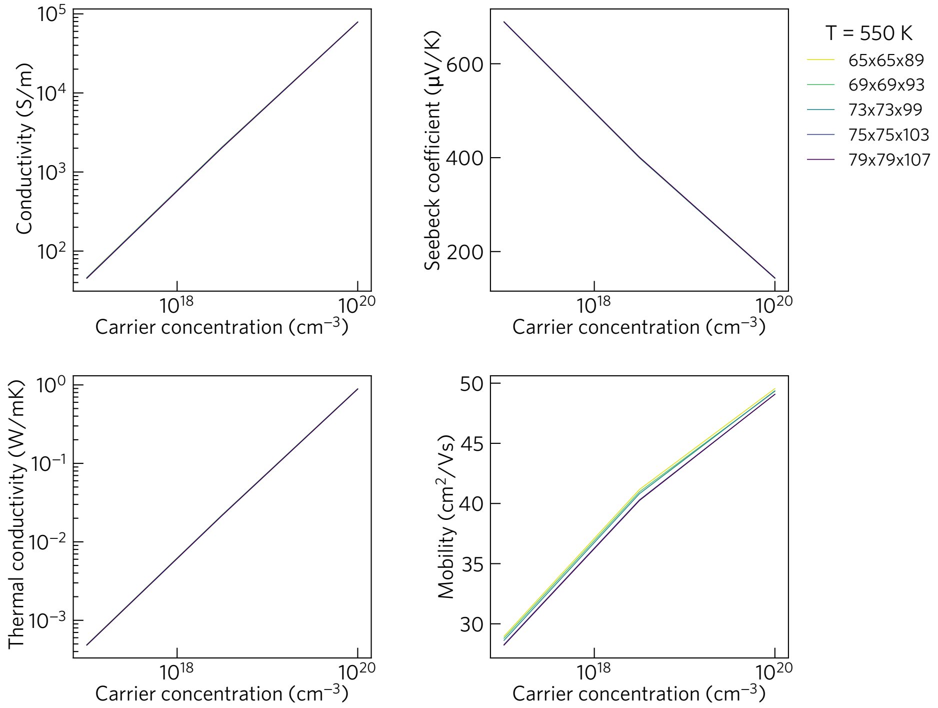

(b)

Figure S2: Electronic transport properties of Ca_4Sb_2O as a function of temperature (a) and carrier concentration (b), calculated over different Fourier interpolated mesh densities.

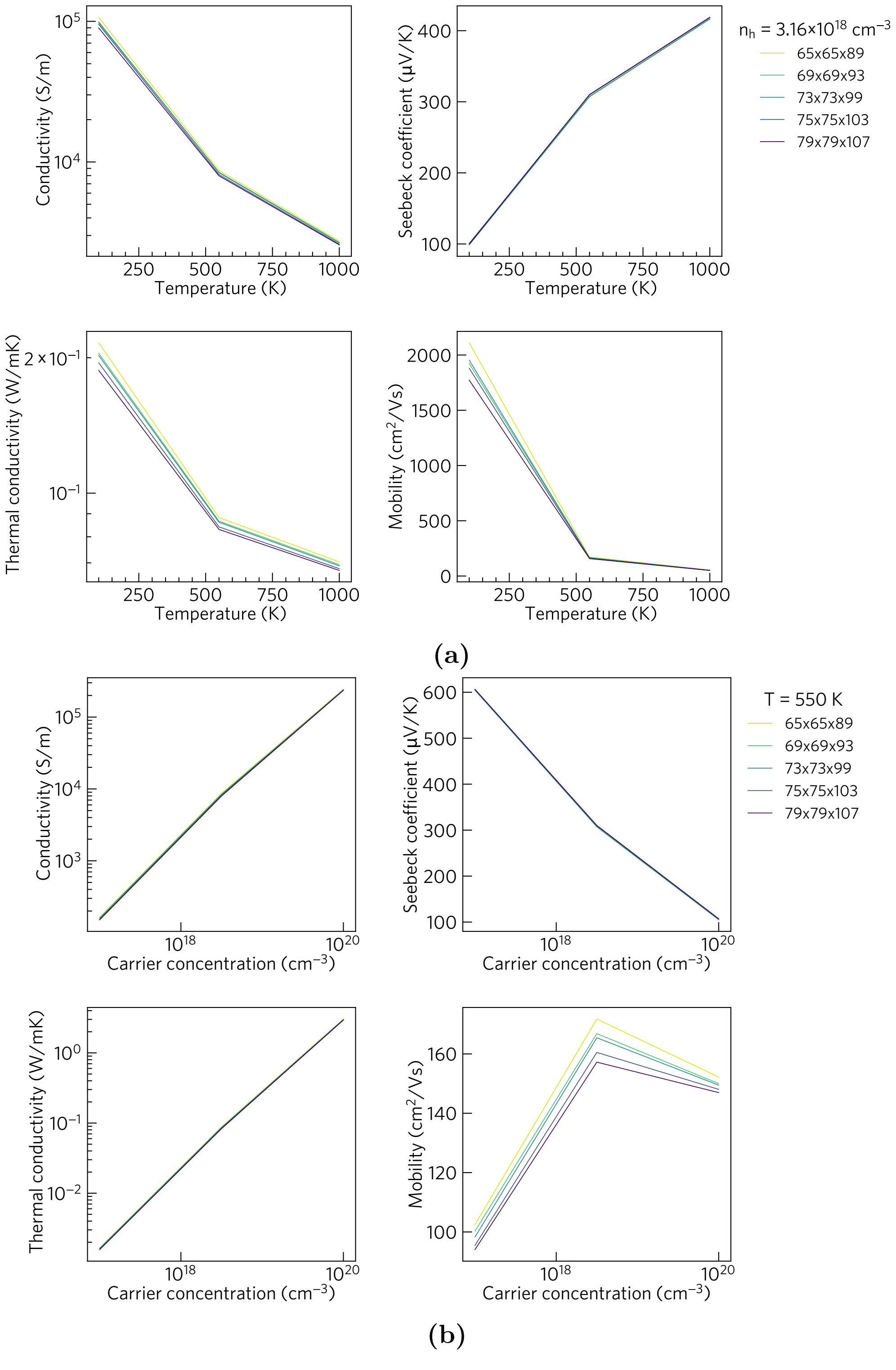

Figure S3: Electronic transport properties of Ca_4Bi_2O as a function of temperature (a) and carrier concentration (b), calculated over different Fourier interpolated mesh densities.

Convergence of the Phonon Frequencies with Respect to Supercell Expansion

The harmonic phonon dispersions of Ca_4Sb_2O and Ca_4Bi_2O were explicitly converged with respect to the supercell expansion used to evaluate the force constants. The tests in Figure S4 show that the phonon dispersion curves are almost converged with a 4×4×1 expansion of the conventional unit cell (224 atoms), but for greater accuracy we opted to use a larger 4×4×4 expansion of the primitive cell (448 atoms).

The third-order interatomic force constants are typically short-ranged compared to the second-order force constants, and we therefore used an 84-atom cubic supercell expansion of the primitive cell to evaluate them. The non-diagonal supercell matrix used to generate this supercell, which is done according to Eq. (1), is shown in Eq. (2):

$$(a_s b_s c_s) = (a_u b_u c_u) M_s \tag{1}$$

where M_s is the supercell matrix, the lattice vectors of unit cell are given by a column vector with components a_u, b_u, c_u, and the vectors of the supercell are given by a vector with components a_s, b_s, c_s.

$$\begin{bmatrix} 2 & 2 & -1 \\ 2 & -2 & -1 \\ 3 & 0 & -3 \end{bmatrix} \tag{2}$$

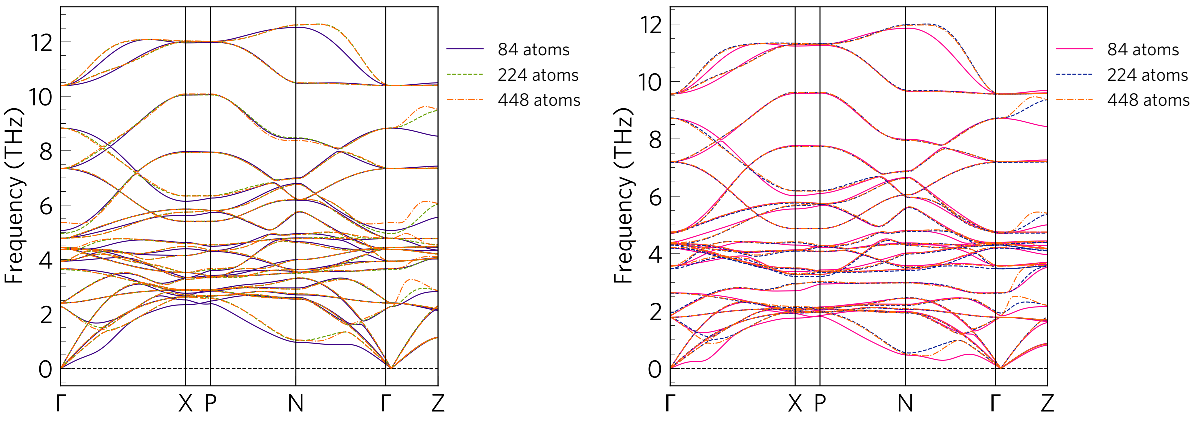

(a) **(b)**

Figure S4: Harmonic phonon dispersion curves of Ca_4Sb_2O (a) and Ca_4Bi_2O (b) obtained using a range of supercell expansions to calculate the second-order force constants.

Convergence of κ_l with Respect to q-Point Sampling Mesh

The changes in the principal xx, yy and zz components of κ_l tensors and the isotropic average $\kappa_{iso} = \frac{1}{3}(\kappa_{xx} + \kappa_{yy} + \kappa_{zz})$ at $T = 300$ K, obtained using different q-point sampling meshes, are shown in Figure S5. The κ_l of Ca_4Sb_2O converges with a 15×15×15 mesh, which produces a κ_{iso} within ≈ 1 % of that obtained using a smaller 13×13×13 mesh. The convergence of Ca_4Bi_2O is erratic, but the values obtained with a 15×15×15 mesh appear to be compatible with the values that larger meshes with up to 40×40×40 subdivisions converge toward, so we use the same mesh for Ca_4Bi_2O.

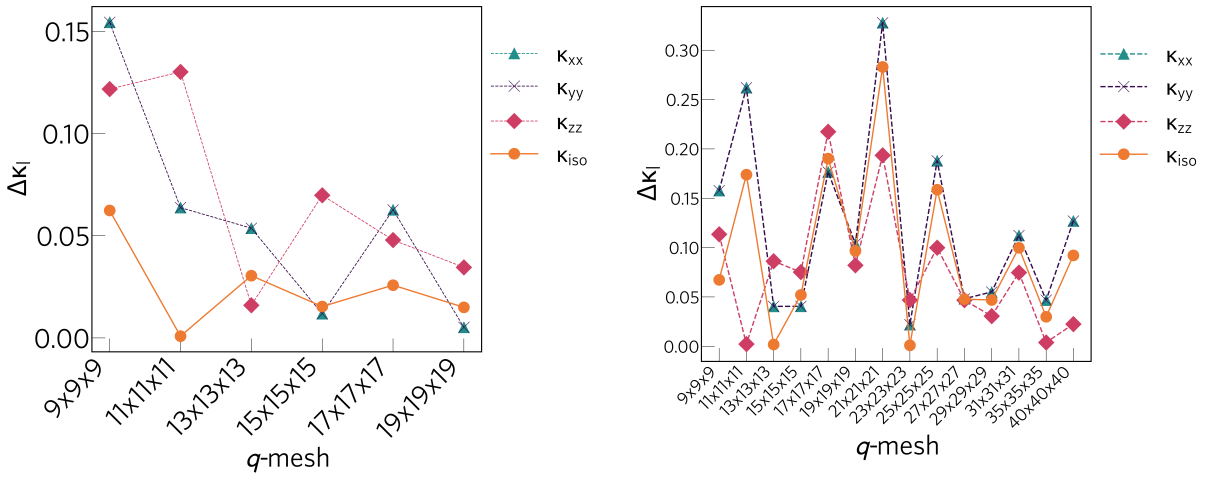

(a) **(b)**

Figure S5: Principal xx (teal triangles), yy (purple crosses) and zz components (pink diamonds) of the κ_l tensor and the isotropic averag κ_{iso} (orange circles) at $T = 300$ K as a function of q-point sampling mesh for Ca_4Sb_2O (a) and Ca_4Bi_2O (b).

Effect of Phonon-Isotope Scattering on κ_l

The presence of isotopes with different masses introduces natural variation at atomic sites that can act as an additional source of phonon scattering. `Phono3py`[2] implements the model described in ref. 3 to estimate this contribution. As shown in Figure S6, we find that natural isotope scattering has a negligible effect on the κ_l of both materials.

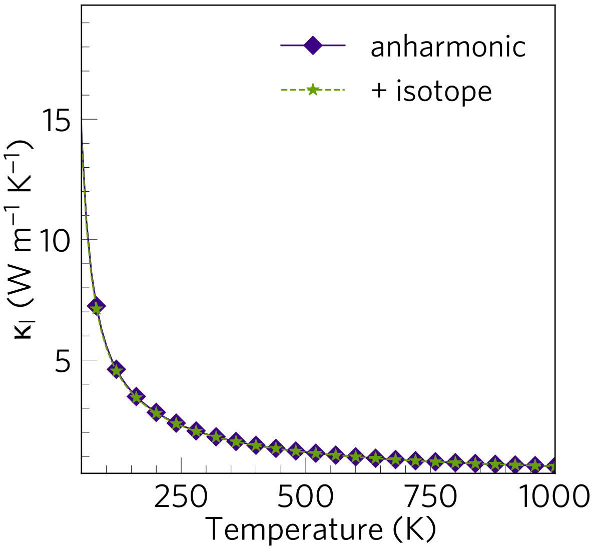

(a)

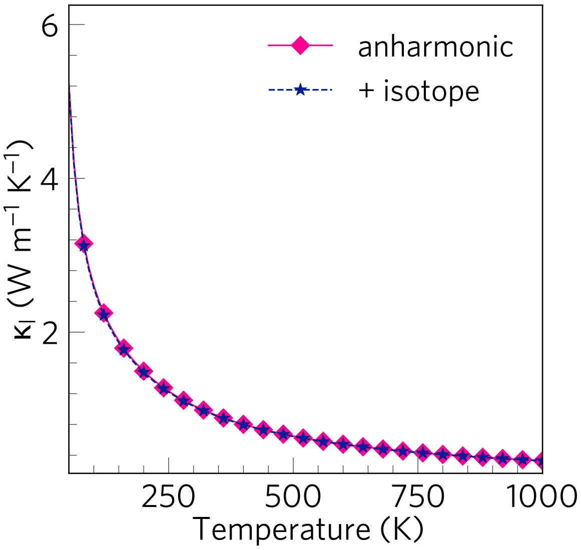

(b)

Figure S6: Lattice thermal conductivity κ_l of Ca_4Sb_2O (a) and Ca_4Bi_2O (b) computed with and without natural isotope scattering.

Anisotropic Cumulative Lattice Thermal Conductivity

Figure S7 shows the cumulative % lattice thermal conductivity as a function of frequency for Ca_4Sb_2O and Ca_4Bi_2O separately along the a/b and c directions. In both materials, the cumulative contributions to κ_l along both directions shows a sharp increase over the acoustic-mode frequencies and a slower rise over the optic-mode frequencies. Therefore, the acoustic modes make the largest contribution to the heat transport along both directions, with a significant further contribution from the optic modes.

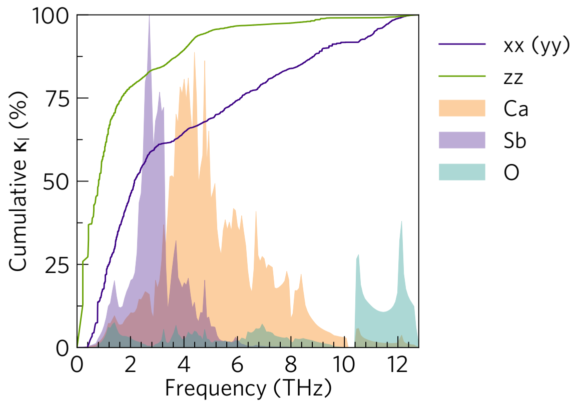

(a)

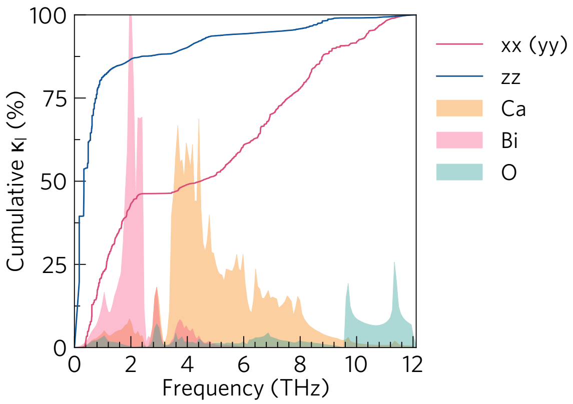

(b)

Figure S7: Cumulative % lattice thermal conductivity of Ca_4Sb_2O (a) and Ca_4Bi_2O (b) as a function of frequency at $T = 300$ K along a/b (purple and pink) and c axes (green and blue). The cumulative κ_l is overlaid against the phonon density of states projected onto Ca (orange), Sb (purple), Bi (pink) and O (cyan) atoms for comparison.

Anisotropic Modal Contributions to the κ_l

As described in the text, due to the tetragonal symmetry of the crystals the lattice thermal conductivities of Ca_4Sb_2O and Ca_4Bi_2O are anisotropic and differ along the a/b and c directions. Figure S8 compares the directional group velocity norms $|\nu_\lambda|$ and mean free path norms $|\Lambda_\lambda|$ of each material along the in-plane and out-of-plane directions.

(We note that for this comparison we compute ν_λ from $\sqrt{\mathrm{Tr}[\nu_\lambda \bigotimes \nu_\lambda]}$. `Phono3py` outputs ν_λ at irreducible q-points which, depending on symmetry, may not be representative of the group velocities along directions. However, the outer products $\nu_\lambda \bigotimes \nu_\lambda$ output by `Phono3py` are summed over symmetry-related q-points and therefore are representative.)

Figure S8 shows that for both structures the maximum ν_λ along the c direction are higher than along the a/b direction. However, the density of modes with low ν_λ in the c direction is much larger, and thus the average velocity along this direction is smaller. Similarly, there is a much higher density of modes with short mean free paths along the c direction compared to the a and b directions. The lower average ν_λ and Λ_λ along the c direction compared to the a/b direction leads to lower out-of-plane κ_l in both materials.

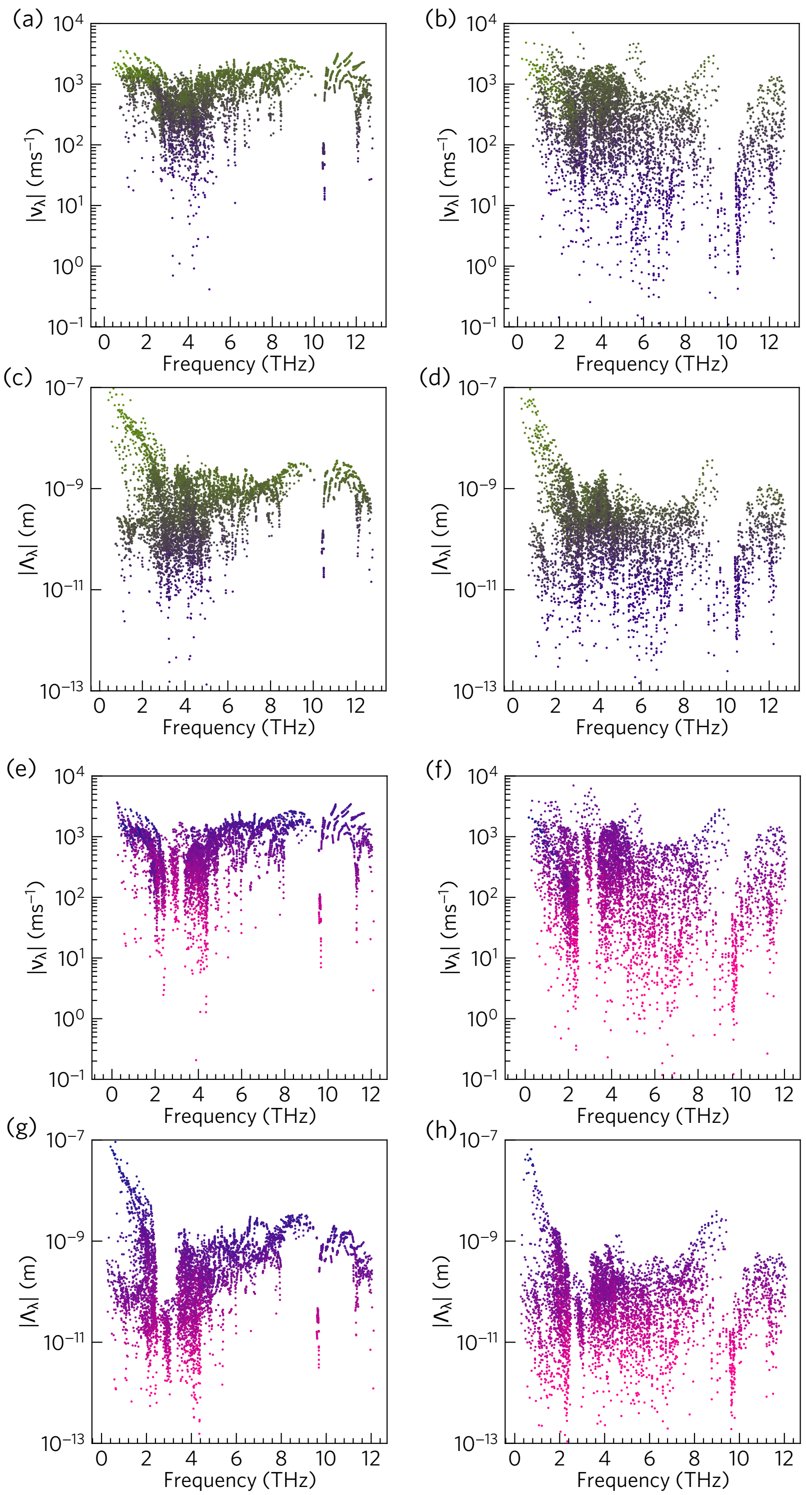

Figure S8: Frequency spectra of the anisotropic modal group velocity norms $|\nu_\lambda|$ (a, b, e, f) and mean free path norms $|\Lambda_\lambda|$ (c, d, g, h) at $T = 300$ K in Ca_4Sb_2O (a-d) and Ca_4Bi_2O (e-h). The left-hand columns show data for transport in the a/b directions and the right-hand column shows data for transport along the c axis. The data points are colour coded by the modal contributions to κ_l, κ_λ, from purple to green (low to high κ_λ) for Ca_4Sb_2O and pink to blue (low to high κ_λ) for Ca_4Bi_2O.

References

(1) Ganose, A. M.; Park, J.; Faghaninia, A.; Woods-Robinson, R.; Persson, K. A.; Jain, A. Efficient Calculation of Carrier Scattering Rates from First Principles. *Nat. Commun.* **2021**, *12*, 2222.

(2) Togo, A.; Chaput, L.; Tanaka, I. Distributions of Phonon Lifetimes in Brillouin Zones. *Phys. Rev. B* **2015**, *91*, 094306.

(3) Tamura, S. I. Isotope Scattering of Dispersive Phonons in Ge. *Phys. Rev. B* **1983**, *27*, 858.
